# A neuromechanical model for *Drosophila* larval crawling based on physical measurements

**DOI:** 10.1186/s12915-022-01336-w

**Published:** 2022-06-15

**Authors:** Xiyang Sun, Yingtao Liu, Chang Liu, Koichi Mayumi, Kohzo Ito, Akinao Nose, Hiroshi Kohsaka

**Affiliations:** 1 Department of Complexity Science and Engineering, Graduate School of Frontier Science, the University of Tokyo, 5-1-5 Kashiwanoha, Kashiwa, Chiba 277-8561 Japan; 2 Department of Physics, Graduate School of Science, the University of Tokyo, 7-3-1 Hongo, Bunkyo-ku, Tokyo, 133-0033 Japan; 3 Department of Advanced Materials Science, Graduate School of Frontier Science, The University of Tokyo, 5-1-5 Kashiwanoha, Kashiwa, Chiba 277-8561 Japan; 4 Division of General Education, Graduate School of Informatics and Engineering, The University of Electro-Communications, 1-5-1, Chofugaoka, Chofu, Tokyo, 182-8585 Japan

**Keywords:** Biomechanics, Viscoelasticity, *Drosophila* larvae, Neuromechanical model

## Abstract

**Background:**

Animal locomotion requires dynamic interactions between neural circuits, the body (typically muscles), and surrounding environments. While the neural circuitry of movement has been intensively studied, how these outputs are integrated with body mechanics (neuromechanics) is less clear, in part due to the lack of understanding of the biomechanical properties of animal bodies. Here, we propose an integrated neuromechanical model of movement based on physical measurements by taking *Drosophila* larvae as a model of soft-bodied animals.

**Results:**

We first characterized the kinematics of forward crawling in *Drosophila* larvae at a segmental and whole-body level. We then characterized the biomechanical parameters of fly larvae, namely the contraction forces generated by neural activity, and passive elastic and viscosity of the larval body using a stress-relaxation test. We established a mathematical neuromechanical model based on the physical measurements described above, obtaining seven kinematic values characterizing crawling locomotion. By optimizing the parameters in the neural circuit, our neuromechanical model succeeded in quantitatively reproducing the kinematics of larval locomotion that were obtained experimentally. This model could reproduce the observation of optogenetic studies reported previously. The model predicted that peristaltic locomotion could be exhibited in a low-friction condition. Analysis of floating larvae provided results consistent with this prediction. Furthermore, the model predicted a significant contribution of intersegmental connections in the central nervous system, which contrasts with a previous study. This hypothesis allowed us to make a testable prediction for the variability in intersegmental connection in sister species of the genus *Drosophila*.

**Conclusions:**

We generated a neurochemical model based on physical measurement to provide a new foundation to study locomotion in soft-bodied animals and soft robot engineering.

**Supplementary Information:**

The online version contains supplementary material available at 10.1186/s12915-022-01336-w.

## Background

Animal behaviour requires both neural circuits and the body. Specific neural circuits referred to as central pattern generators (CPGs) are capable of creating spatiotemporal activity patterns for behaviours [1–5]. CPGs regulate motor neuron activity and muscular contraction to generate force for movement. On the other hand, the body’s physical properties and constraints are also significant factors to guarantee animal movements [6–9]. Accordingly, the integration of neural circuits and body mechanics, referred to as neuromechanics, is one of the essential approaches to revealing motor control mechanisms [10–15].

Soft-bodied animals locomote by deforming their stretchable bodies [16]. Compared to animals with hard skeletons, soft-bodied animals possess high flexibility and can move adaptively in complicated environments [17]. This flexibility of soft-bodied animals suggests that output from neural circuits is not the sole determinant for locomotion. Animal’s physical properties, such as stiffness and viscosity, should also be involved in the dynamics of locomotion [9, 15] and have been measured experimentally [18–23]. However, it is still challenging to reproduce locomotion in soft-bodied animals by mathematical models based on physical measurements. To tackle this issue, we used *Drosophila melanogaster* (*Drosophila*, hereafter) larvae as a model and attempted to build a neuromechanical model describing their crawling behaviour.

Forward crawling is the most predominant mode in fly larval locomotion [24, 25]. By propagating segmental contraction from the posterior to anterior segments, larvae move forward [26–28], and neural circuits for crawling have been intensively examined [29–37]. Previous simulation studies have succeeded in building models describing the propagative nature of crawling behaviour qualitatively [10, 38–40]. In contrast, the physical properties of larvae remain to be studied, and quantitative reproduction of crawling kinematics has not been achieved yet.

Here, we present a neuromechanical model to describe fly larval crawling behaviour based on their locomotion kinematics and soft-bodied biomechanics. First, we recorded forward crawling in the third-instar larvae whose segmental boundaries were labelled by a fluorescent protein and extracted kinematics parameters of crawling. Then, using a tensile tester combined with optogenetics, we measured larval contraction force. Furthermore, larval viscoelasticity was measured by the tensile tester. The measurement indicated that the properties of the larval body were described better by the standard linear solid (SLS) model than the Kelvin-Voigt model previously used [10, 41]. By incorporating these physical parameters with a neural circuit model built previously [10], we established a neuromechanical model for larval locomotion. By optimizing the neural circuit model parameters, we succeeded in building a neuromechanical model that reproduced the kinematics parameter extracted from larva measurements. This model could also reproduce optogenetic studies reported previously. In addition, crawling in a low-friction condition was simulated, and its prediction was confirmed experimentally by analysing floating larvae. Perturbation analyses in our model predicted the importance of intersegmental connections in speed control, contrasting with previous studies [10, 40]. Based on our simulation results, we predicted the intersegmental connectivity in sister species in the genus *Drosophila*. Our model for larval crawling based on physical measurements provides a new approach to locomotion research in soft-bodied animals and soft robot engineering.

## Results

### Measurement of forward crawling behaviour in third-instar larvae

To reveal the physical mechanisms behind *Drosophila* larval crawling, we examined the kinematics of the behaviour and physical properties of the body. We used third-instar larvae because their size (3.53 ± 0.12 mm, *n* = 9 larvae) was large enough to measure physical properties as a soft material. While previous studies examined first- or second-instar *Drosophila* larvae and characterized the segmental kinematics [26, 27, 37, 42, 43], crawling behaviour in the third-instar larvae had not been investigated at a segmental scale but instead at a scale of the entire body [24, 25, 28, 32, 44]. Thus, we examined the segmental dynamics in freely moving third-instar larvae.

**Fig. 1:**
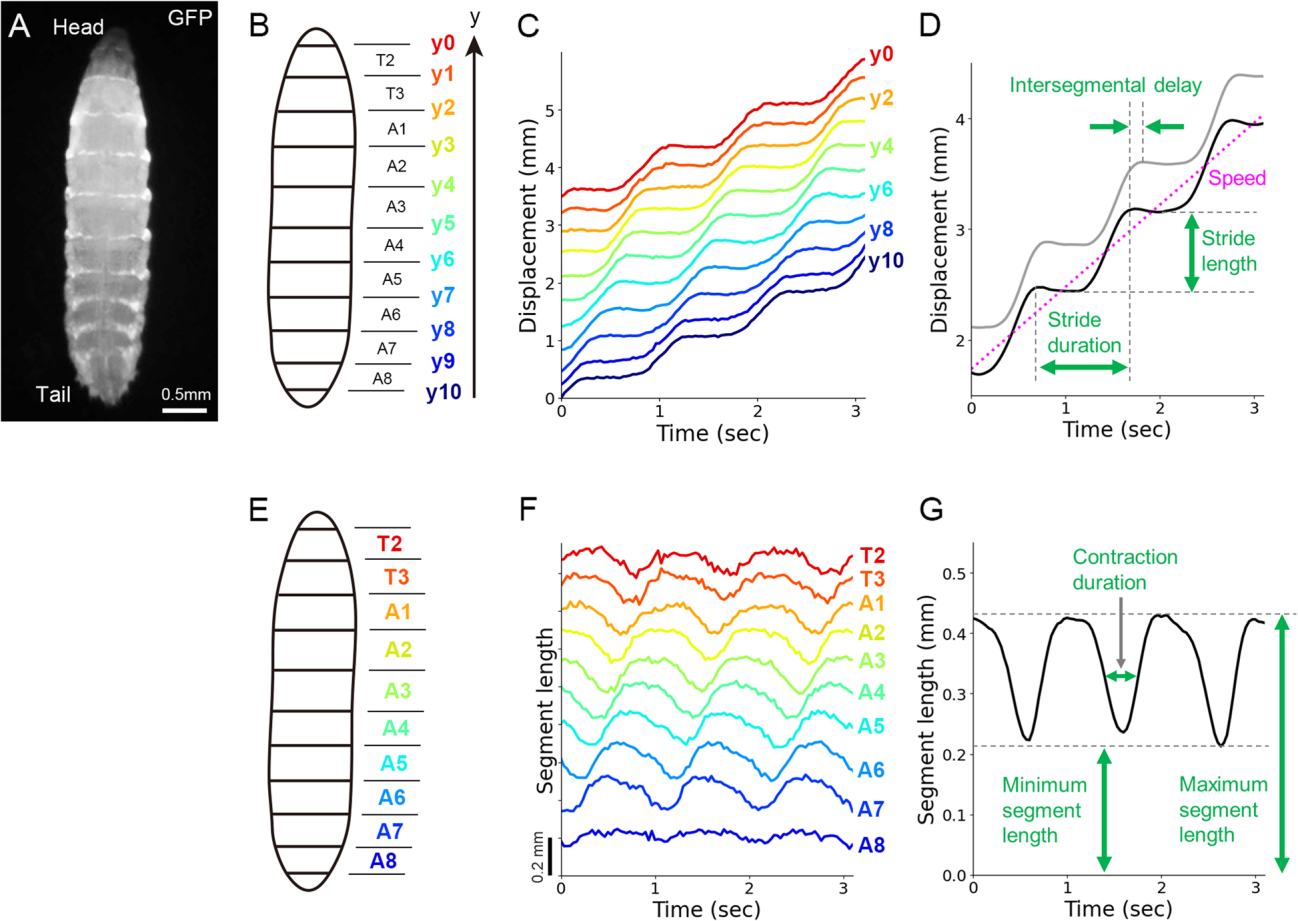
Characterization of forward crawling in third-instar larvae. **A** A fluorescence image of a third-instar larva with the anterior to the top. The segmental boundaries were visualized using *tubP-Gal4, UAS-fondue::GFP*. **B** A schematic of the segment boundaries (y0–y10) and the segment names (T2–A8). **C** Displacement of the segment boundaries during larval crawling. **D** Kinematics parameters based on segmental boundary dynamics. **E** A schematic of the segment names. **F** Segment length changes during larval crawling. **G** Kinematics parameters in segmental length change

First, we recorded the position of the segment boundaries in freely crawling larvae. To reliably trace the segmental boundaries of larvae, we expressed a green fluorescent protein (GFP)-tagged coagulation protein Fondue, which accumulates at the muscle attachment sites [45]. We acquired time-lapse fluorescence images from the dorsal side of the larvae, where longitudinal muscles span single segments (Fig. 1A). From these fluorescence images, the segmental boundaries of the thoracic (T2–T3) and abdominal (A1–A8) segments were annotated (Fig. 1B; see the “Methods” section for details.) We examined the displacement of each segment boundary during forward crawling. As previously reported in the first- and second-instar larvae cases, sequential displacement of the segmental boundary from the posterior to anterior segments was observed in third-instar larvae (Fig. 1C). Four kinematic parameters were measured from the recording: stride length, stride duration, intersegmental delay, and speed (Fig. 1D). We obtained that stride length was 0.70 ± 0.05 mm, stride duration was 1.07 ± 0.12 sec, intersegmental delay was 0.09 ± 0.01 s, and speed was 0.64 ± 0.02 mm/s (*n* = 9 larvae).

Next, we quantified the dynamics of segmental length change. Plots of the segmental length showed the propagation of segmental contraction in crawling larvae (Fig. 1E, F). We measured three kinematic parameters (maximum segment length, minimum segment length, and contraction duration described in Fig. 1G) and obtained that maximum segmental length was 0.41 ± 0.03 mm, minimum segmental length was 0.21 ± 0.02 mm, and contraction duration was 0.44 ± 0.03 s (*n* = 9 larvae). To sum, we measured the seven kinematics quantities from segmental dynamics in freely crawling GFP-labelled third-instar larvae.

### Consistent kinematic properties among segments in larval crawling

**Fig. 2:**
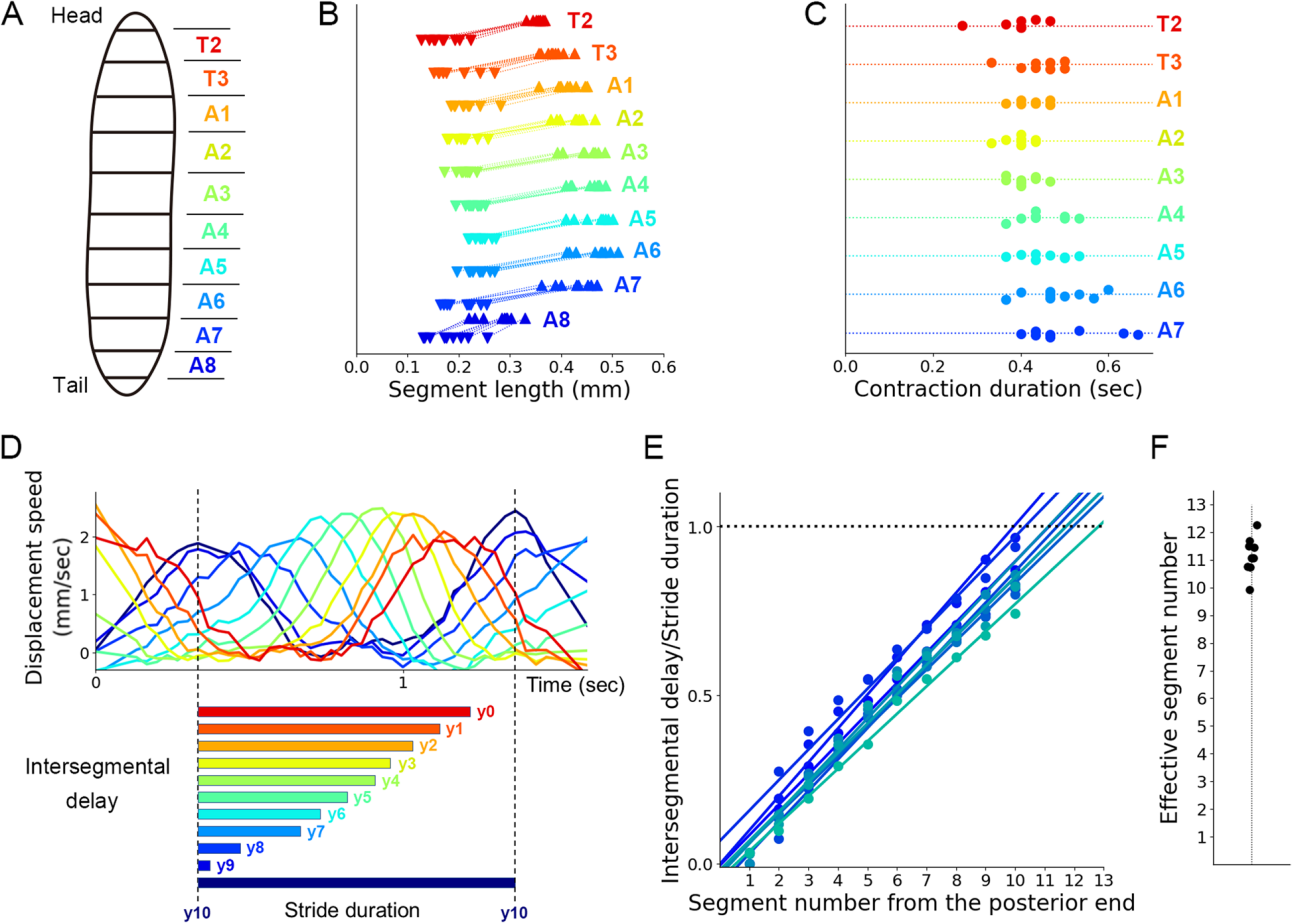
Consistent kinematics properties among segments in larval crawling. **A** A schematic of the segment names. **B** The range of segment length changes during larval crawling. Upward and downward triangles show the maximum and minimum length of the segments, respectively. **C** Contraction duration in larval segmental dynamics. **D** Displacement speed of larval segmental boundaries and schematics of their intersegmental delay. **E** Linear regression for larval intersegmental delays. **F** Effective segment numbers estimated by linear regression in (**E**)

To establish a mathematical model for larval crawling, we examined whether we could assume that all the segments possess similar kinematic properties or not. To this aim, we compared the dynamics of segmental length change (defined in Fig. 1G) among all the segments (Fig. 2A–C). We noticed that the most posterior A8 segment had a shorter maximum length and smaller contraction range than the other segments (Fig. 2B). This observation reflected the fact that the A8 segment was a specialized structure and different from the others in the surface area of the body wall and the number of muscles [46]. In contrast, in the other segments, both the minimum and maximum lengths were comparable over the segments (Fig. 2B). Furthermore, contraction duration was also similar among the segments from T2 to A7 (Fig. 2C). Accordingly, most segments (T2–A7) exhibited similar kinematic dynamics in contraction during crawling, which allowed us to model the larva as a chain of segments with the same kinematic properties.

Having confirmed the similarity among the body segments, we inferred the effective number of segments. First, we adopted a segment model based on the “piston model” of larval peristalsis, where the motion of the head and tail were coupled [10, 26]. When a peristaltic wave reached the head, the tail was forced to move forward, which induced the contraction of the posterior segment A8 for the next stride of crawling. Then, we estimated the effective number of segments by assuming that the displacement of the virtual anterior end was the same as that of the tail. To evaluate the propagation velocity, we obtained intersegmental delay by calculating the cross-correlation of displacements between the most posterior segment boundary (y10) and the other segments (y0–y9) (Fig. 2D). The graph showed the propagation of segmental contraction from the posterior to the anterior segments. By plotting the intersegmental delay normalized by the stride duration, we noticed that the segmental contraction propagates at about a constant speed (Fig. 2E). This observation allowed us to model the larval crawling as the propagation of segmental contraction at a uniform speed. Based on this assumption, we inferred the effective number of segments of the whole body where the head and tail were coupled. To this aim, we fitted the intersegmental delay data by linear regression. The intersection points between the fitting lines and the horizontal line with a value of one on the vertical axis corresponded to the event when the propagation of segmental contraction reached the anterior end, and the subsequent propagation was initiated. From this calculation, we obtained 11.2 ± 0.2 as the effective number of segments (Fig. 2F, *n* = 9 larvae). Accordingly, the results indicated that the crawling larva could be modelled by a chain of 11 identical segments (Additional file 1: Fig. S1).

### Measurement of larval contraction force

**Fig. 3:**
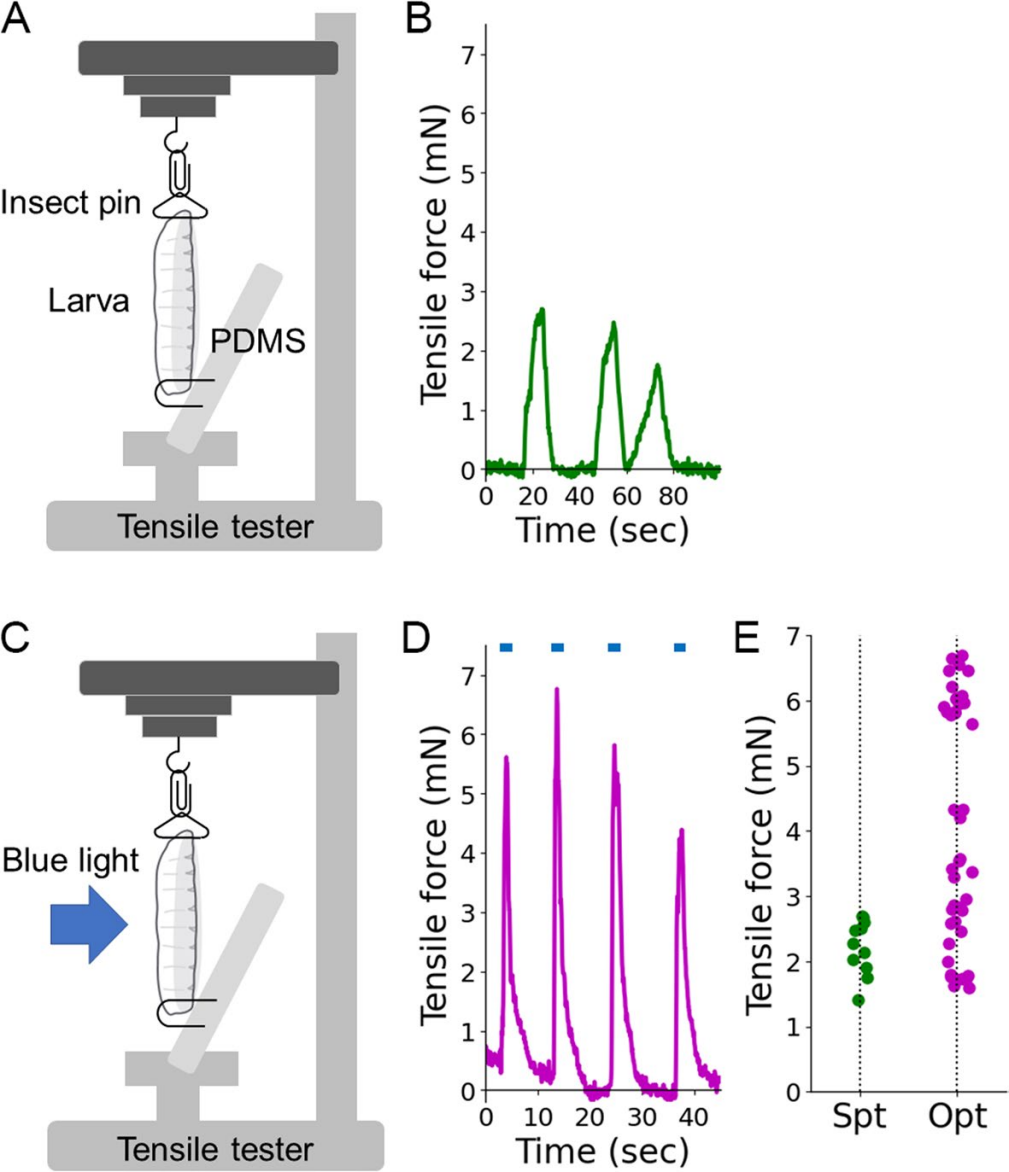
Measurement of contraction force in a larva. **A** A larva was hooked at the head and tail. The extension and tensile force of the larva were measured by a tensile tester. **B** A sample trace of spontaneous contraction force of a larva. **C** Optogenetic stimulation to a larva during measurement with the tensile tester. **D** A sample trace of larval force with optogenetic activation (shown in blue bars above the trace). **E** The tensile force of larvae generated by spontaneous contraction (“Spt”; *n* = 11 contraction events from three larvae) and optogenetic activation (“Opt”; *n* = 44 activations from seven larvae)

To establish a quantitative physical model for larval crawling, we attempted to measure the contraction force and soft material properties of larvae which enabled us to derive a Newtonian description of crawling by linking force to motion. We measured contraction force with the tensile tester (Fig. 3A). The tensile tester allowed us to measure and control the strain and stress of a sample material. We hooked an intact larva with insect pins and loaded it to the tensile tester. We measured the spontaneous contraction force in larvae and obtained values ranging from 1.4 to 2.7 mN (*n* = 11 spontaneous contraction events by three larvae, Fig. 3B, E). Although these values reflected the range of spontaneous contraction force, the larvae could not exhibit crawling locomotion during the measurement since their head and tail were hooked. Accordingly, we instead attempted to measure the maximum force that larvae could generate for crawling. To this aim, we adopted optogenetics to activate motor neurons reliably (Fig. 3C). We expressed a light-sensitive cation channel protein Channelrhodopsin2 [47] in all of the larval type-I motor neurons [48] and illuminated each larva with blue light (455 nm, 5.7 nW/mm^2^) to induce the contraction of all the body wall muscles. Since we needed to measure the muscular force evoked by the central nervous system (CNS), we expressed Channelrhodopsin2 in motor neurons instead of the muscles. The contraction force measurement with the optogenetic activation showed that the force ranged from 1.6 to 6.7 mN (*n* = 44 optogenetic stimulations applied to seven larvae, Fig. 3D, E). The variation in the measurement would be caused by the differences in some uncontrollable conditions, including hooking states at the head and tail. Consequently, we adopted the maximum measured force of 6.7 mN as the maximum force (${F}_{M_{\mathrm{max}}}$) used in our neuromechanical model.

### Measurement of elastic and damping properties of larvae

**Fig. 4:**
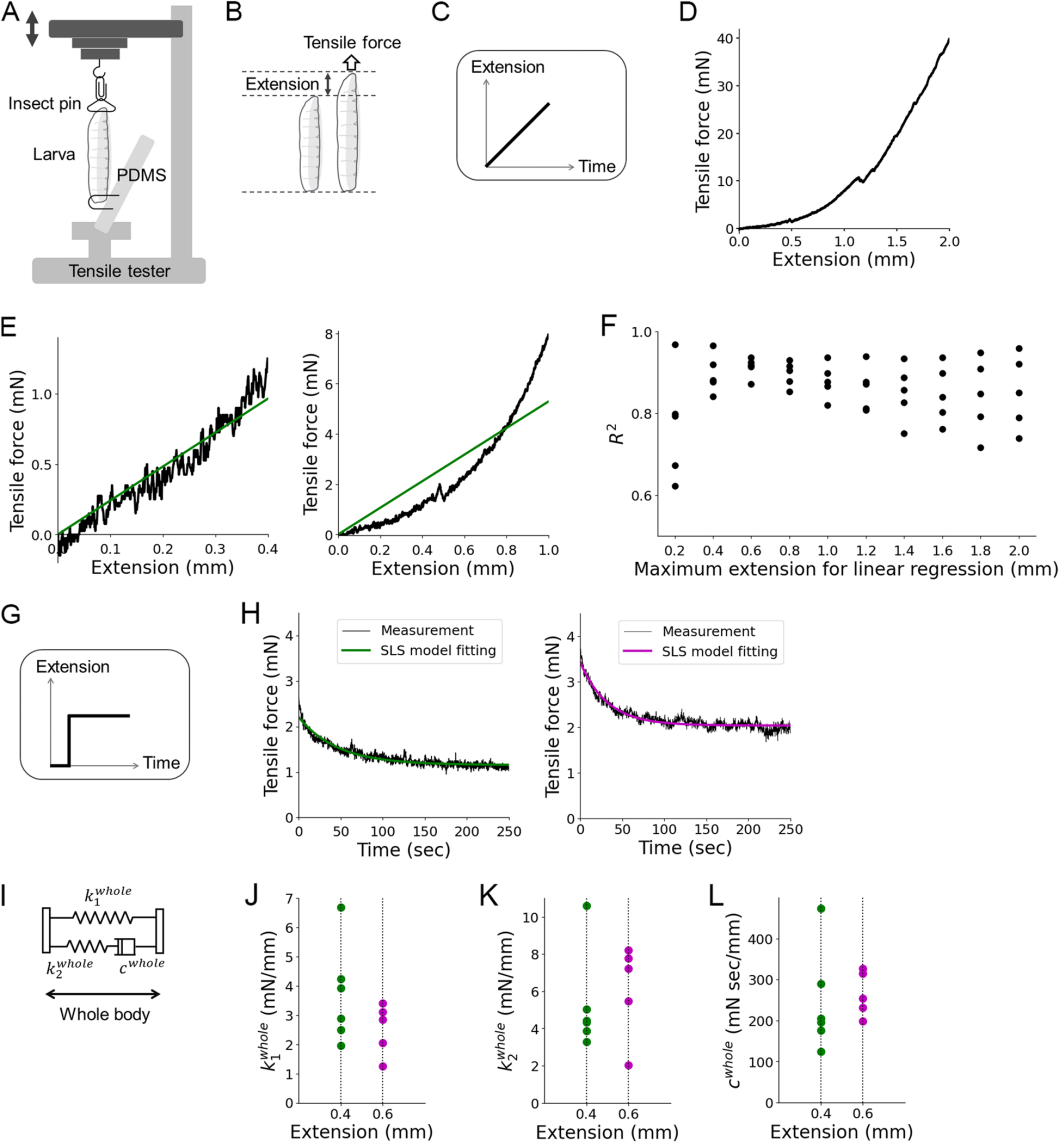
Measurement of viscoelasticity of the larval body. **A** Measurement and control of extension and tensile force of larval body with the tensile tester. The double-headed arrow indicates that the extension is changeable during the measurement. **B** A schematic of tensile force and extension of a larva. The left larva is not subjected to external forces while the right one is extended. The applied force to extend is the tensile force. **C** In the measurement of the relationship between extension and tensile force, the larvae were extended at a constant rate as shown in this panel, and the tensile force is being recorded. **D** An example trace of the relationship between extension and tensile force. **E** Plots of **D** in two different ranges of extension (left 0–0.4 mm, right 0–1.0 mm). Green lines indicate linear regression lines in these ranges. **F** The coefficient of determination of linear regression in different ranges of extension. **G** In the stress-relaxation test, the larvae were extended quickly, and their length was kept constant. The tensile force under the constant extension was being recorded. **H** Example traces of stress-relaxation tests with the constant strain of 0.4 mm (left) and 0.6 mm (right). Fitting curves with the SLS model are shown in green (left) and magenta (right). **I** By the SLS model, the whole larval body can be described as two springs (spring constants: ${k}_1^{\mathrm{whole}}$ and ${k}_2^{\mathrm{whole}}$) and one damper (damping coefficient: *c*^whole^). **J**–**L** Scatter plots of ${k}_1^{\mathrm{whole}}$ (**J**), ${k}_2^{\mathrm{whole}}$ (**K**), and *c*^whole^ (**L**) measured with the maximum strain of 0.4 mm (green) and 0.6 mm (magenta)

Next, we analysed the passive properties of the larval body, elasticity, and viscosity, with the tensile tester (Fig. 4A). The stretchable body wall and hydrostatic skeleton of fruit fly larvae were typical components in soft-bodied organisms [49]. Previous studies reported material properties of soft-bodied animals, including the caterpillar *Manduca sexta* [18] and the earthworm *Lumbricus terrestris* [50, 51]. Although previous theoretical analyses of fly larval locomotion assumed that each segment was equivalent to a pair of a spring (a linear elastic component) and a damper (a linear viscous component) [10, 39, 41], the mechanical properties of *Drosophila* larvae were not measured experimentally. Accordingly, we measured the viscoelastic properties of fly larvae by methods used in material science.

First, we analysed the range of extension within which we could assume linearity between the extension and tensile force of the larval body (Fig. 4B). To analyse the relationship between the extension and tensile force of larvae, we stretched the larvae at a constant speed (Fig. 4C) and plotted tensile force against the extension (Fig. 4D). To test the linearity, we used linear regression. Depending on the range of extension, the goodness of fitting varies: linear regression fitted better in the range from 0 to 0.4 mm extension (Fig. 4E, left) than in a longer range from 0 to 1.0 mm (Fig. 4E, right). To find a suitable extension range with good linearity, we calculated the coefficient of determinants, which was an indicator of the predictability of the data by linear regression (Fig. 4F). The plot showed that extensions up to 0.4 mm or 0.6 mm exhibited high coefficients. Based on this observation, we concluded that we could assume linearity in the stretch ranging from 0 to 0.6 mm.

Next, we measured the viscoelasticity of larvae. Viscoelastic properties of soft materials could be acquired by measuring the relationship between stress (force loaded in the material) and strain (deformation of the material) [52]. To obtain the viscoelastic properties of larvae, we conducted the stress relaxation test, one of the standard dynamic mechanical tests [52]. In this test, a soft material sample was quickly extended to a certain length, and then the stress was being recorded over time (Fig. 4G). Based on the linearity test above, we extended the third-instar larvae by 0.4 mm. The result of the stress relaxation test showed an exponential decay that was approaching a non-zero plateau at a later time (Fig. 4H, left). To extract the physical properties from the stress relaxation data, a suitable physical model to describe the larval body was required. Among the general mechanical structure models of linear combinations of springs and dampers, we found that the Maxwell model and the Kelvin-Voigt model, which were used previously to model larval musculature [10, 39], could not fit the experimental curve well since either exponential decay or residual elastic force was missing in these models (Additional file 2: Fig. S2). In contrast, the standard linear solid (SLS) model, which combined the Maxwell model and a Hookean spring in parallel [52] (Fig. 4I), fitted the experimental results well. Thus, we fitted this curve with the general SLS model (Fig. 4H, left). Based on the SLS model, we obtained the two spring constants and one damping coefficient for the whole body of intact larvae by the extension of 0.4 mm (${k}_1^{\mathrm{whole}}$ = 3.7 ± 0.7 mN/mm, ${k}_2^{\mathrm{whole}}$ = 5.3 ± 1.1 mN/mm, *c*^whole^ = (2.4 ± 0.5) ×10^2^ mN s/mm, from *n* = 6 larvae, Fig. 4J–L). Consistent values were obtained by the extension of 0.6 mm (${k}_1^{\mathrm{whole}}$ = 2.5 ± 0.4 mN/mm, ${k}_2^{\mathrm{whole}}$ = 6.1 ± 1.1 mN/mm, *c*^whole^ = (2.6 ± 0.2) ×10^2^ mN s/mm, from *n* = 5 larvae, Fig. 4H right J–L), which further supported the linear property of larvae. The two spring constants did not have a strong correlation (*r* = 0.49, *p* = 0.13, *n* = 11; Pearson’s correlation coefficient) suggesting that they could not be reduced to a single spring constant. To sum, we obtained the viscoelastic parameters of larvae, two spring constants and one damping coefficient, by the stress relaxation test. It should be noted that in larval crawling, each segment exhibited contraction instead of extension which we induced in the measurement. However, we could not measure the viscoelasticity of larvae during contraction since larvae were bent instead of compressed when pushed from the both ends. Accordingly, we assumed larvae possess the same linear viscoelastic properties both in extension and compression. It should also be noted that in larval crawling, each segment contracted by about 0.2 mm (Fig. 2B) while in the stress-relaxation experiment, each segment was extended by about 0.04 mm (0.4 mm/11 segments). There could be some non-linearity in the viscoelastic properties of the larval body. However, in our model, we adopted the viscoelasticity measured with 0.4-mm extension as the approximate values for our mathematical model of larval crawling.

### A neuromechanical model for larval crawling referring to the physical measurement

**Fig. 5:**
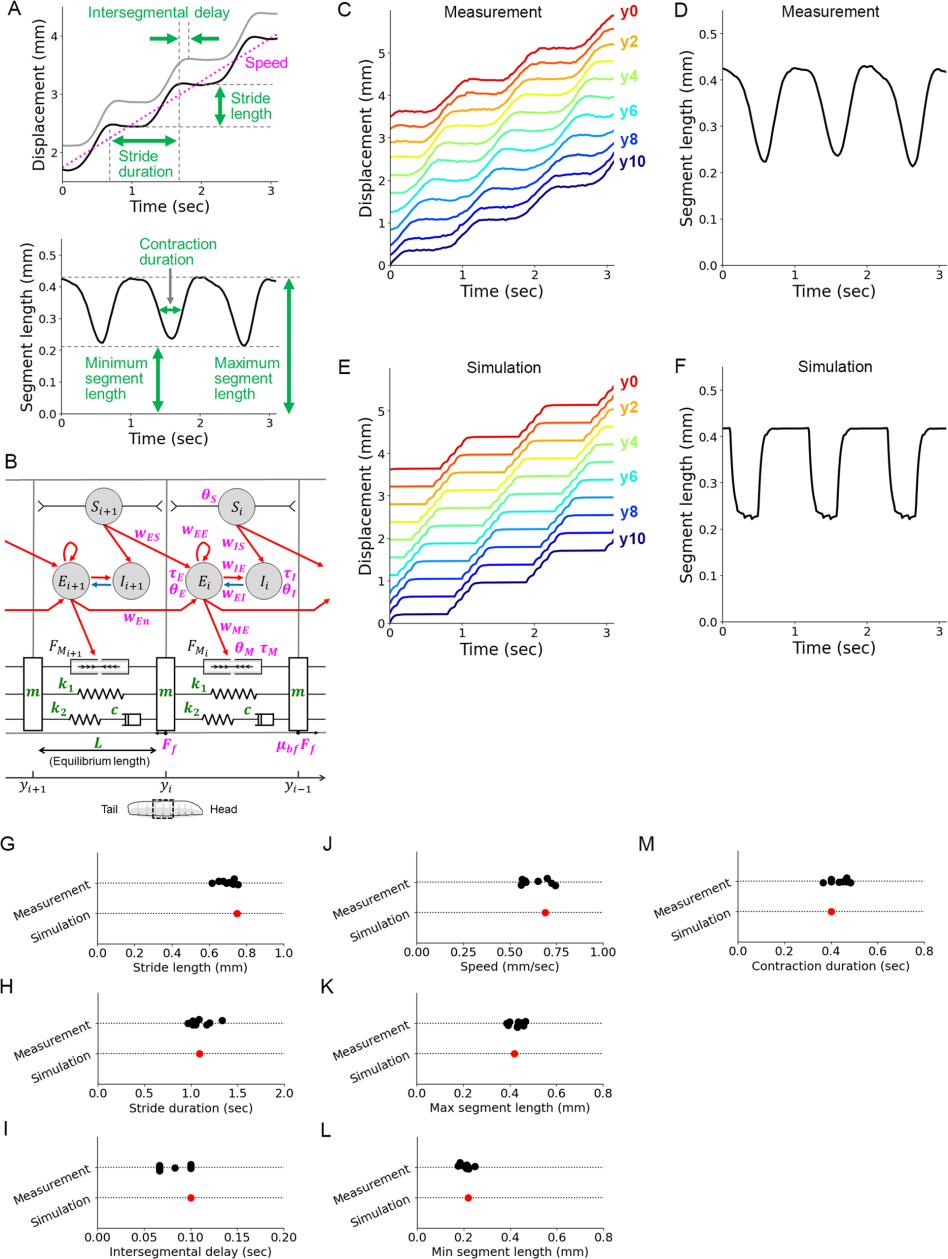
Reproduction of larval crawling by a neuromechanical model. **A** Kinematics parameters in segmental boundary dynamics (left, same as Fig. 1D) and segmental length change (right, same as Fig. 1G). **B** A schematic of our neuromechanical model for larval crawling. The neural circuit was referred to Pehlevan et al. [10], although the parameter values in the model were different. Since we assumed *w*_ES_ = *w*_IS_ as stated in Pehlevan et al. [10], the total number of the free parameters (written in magenta) was 15. Two segments in a larva (corresponding to the dotted box below) are drawn. Circles are populations of neurons: *E*_*i*_ is an excitatory neuron group, *I*_*i*_ is an inhibitory neuron group, and *S*_*i*_ is a sensory neuron group in the *i*th segment. Excitatory and inhibitory connections are labelled by red and blue arrows, respectively. The body of the *i*th segment is modelled by two springs (*k*_1_ and *k*_2_), one damper (*c*), and one muscle whose force is *M*_*i*_. Segment boundaries have masses (*m*) that feel friction with the substrate (*F*_*f*_ in forward motion and *μ*_*bf*_*F*_*f*_ in backward motion). The position of the *i*th segment is denoted as *y*_*i*_. **C** A kymograph of segmental boundaries during larval crawling (same as Fig. 1C). **D** Change in segmental length during larval crawling (same data shown in Fig. 1G). **E** A simulation result of kymograph of segmental boundaries. **F** Simulation result of segmental length. Segment labels and line colours in **C** and **E** correspond to those in Fig. 1B, C. **G**–**L** Comparison of seven kinematics between parameters obtained from larval crawling (black dots, *n* = 9 larvae) and simulation (red dots). **G** Stride length. **H** Stride duration. **I** Intersegmental delay. **J** Crawling speed. **K** Maximum segment length. **L** Minimum segment length. **M** Contraction duration of single segments

We established a mathematical neuromechanical model based on the physical measurements described above. On the other hand, we obtained seven kinematic values characterizing crawling locomotion (Fig. 5A) from the measurement of fly larvae (Figs. 1 and 2). Our aim was to build a model with a neural circuit and mechanical components to reproduce the observed results quantitatively. Regarding the mechanical part, based on the measurements, we estimated that the larva was a chain of eleven consistent segments (Fig. 2), each of which was described by the SLS model (Fig. 4 and Additional file 1: Fig. S1). These assumptions indicated that the viscoelastic parameters for single segments in the model (two spring constants *k*_1_ and *k*_2_ and one damping coefficient *c*) should be 11 times larger than the ones for the whole body (*k*_1_ = 40.7 mN/mm, *k*_2_ = 58.3 mN/mm, and *c* = 2640 mN s/mm). Furthermore, the force measurement indicated that the maximum muscular contraction force could be 6.7 mN (Fig. 3). We modelled segmental boundaries as masses that were pulled and pushed by the segmental muscles and dragged on the surface with friction (Fig. 5B). We measured the mass of larvae (1.14 mg, the 99% confidence interval is [1.12, 1.15], *n* = 70 larvae) and calculated the mass of one segment by dividing the mass of the whole body by the number of segments to obtain 0.1 mg/segment. The length of larvae was 3.5 ± 0.1 mm (*n* = 9). We used all these values in our mechanical model (green parameters in Fig. 5B). In contrast, for the neural network part, we adopted a neural circuit model built by Pehlevan et al. [10] based on Wilson-Cowan equations. This model was capable of generating propagation of waves from the posterior to anterior segments by interaction among excitatory and inhibitory neurons in the CNS, motor neurons, and sensory neurons [10]. Although the parameters in this model were suggested in the previous paper, we incorporated the neural circuit framework and tuned its parameters to build a neuromechanical model based on physical measurement to reproduce larval crawling.

Aiming to reproduce the measurement results on larval crawling, we adjusted 15 parameters in the neural circuit model (magenta parameters in Fig. 5B). The optimized set of the parameters (Additional file 3: Fig. S3) could reproduce the measurement results of the kinematics (Fig. 5C–F). To assess this observation quantitatively, we compared the seven kinematics parameters (Fig. 5A) between the measurement and simulation and found that all the parameters could be reproduced by the simulation to fit in the ranges of the measurement results (Fig. 5G–M). Accordingly, this neuromechanical model could quantitatively describe the segmental kinematics during forward larvae crawling.

The stress-relaxation results showed large variabilities on the order of 100% of the mean values (Fig. 4J–L). This variance could be caused by the uncertainty in the measurement or the variability in physical properties among larvae. Since we did not have independent measurement methods to obtain viscoelasticity, we could not determine which one was the case. However, when we perturbed the spring constants by the order of 100% of the optimized value, the crawling speed changed to be out of the measured range (Additional file 4: Fig. S4). This observation suggested that the viscoelasticity would be consistent between larvae, and the variabilities in the stress-relaxation test would be caused by uncertainty in the measurement.

We adopted *μ*_*bf*_, the asymmetricity parameter between the forward and backward friction is 10, considering the fact that a larva had denticle belts on the ventral side of the body, providing asymmetric friction with the ground. To test the dependency of crawling on this asymmetricity, we perturbed *μ*_*bf*_ (Additional file 5: Fig. S5). The simulation results showed that crawling speed did not change even when *μ*_*bf*_ equalled 1, meaning that the friction was symmetric between forward and backward. This observation indicated that the asymmetricity in the friction along the body axis had a small role in generating forward crawling.

As for the maximum muscular contraction force, we adopted the maximum force observed by optogenetic stimulation. To test whether we could use smaller muscular forces to reproduce larval crawling, we conducted a perturbation analysis on the maximum muscular force in our neuromechanical model (Additional file 6: Fig. S6). The simulation results showed that crawling speed reduced as the muscular force decreased. Accordingly, this observation suggested that crawling speed was sensitive to the maximum muscular force, and the measurement of contraction force was crucial to building a neuromechanical model for larval crawling.

We compared parameters in our model based on physical measurements with those in the previous simulation by Pehlevan et al. [10]. In Pehlevan et al. [10], parameters were scaled by three values, the spring constant *k*, the equilibrium length of one segment *L*, and the relaxation time scale of the excitatory neurons *τ*_*E*_. Accordingly, we substituted these parameters with our measurement results (*k* = 50 mN/mm, *L* = 0.35 mm, and *τ*_*E*_ = 35 ms) and obtained the absolute values of the parameters. By comparison, we found that the neural couplings and relaxation times in the CNS were almost consistent between our results and the assumption in Pehlevan et al. [10]. On the other hand, physical properties showed clear contrast between them. The damping coefficient in our result (*c* ~ 2640 mN s/mm) was about 400 times larger than the previous assumption [10] (*c* ~ 6 mN s/mm). When we adopted the damping coefficient 400 times smaller than our value, the crawling speed was affected to be doubled (crawling speed 0.69 mm/s in the optimized condition; 1.32 mm/s when the damping coefficient c was 400 times smaller than the optimized value of *c*, Additional file 4: Fig. S4C), and the maximum muscle contraction force in our results (*F*_*M*max_ = 6.7 mN) was 2.2 times smaller than the previous assumption [10] (*F*_*M*max_ = 15 mN). In addition, neural couplings between the CNS and either muscles or sensory neurons also exhibited differences: The coupling between the excitatory neurons and muscles in our model (*w*_ME_ = 540) was larger the previous assumption (*w*_ME_ = 1 in [10]), and the couplings between the sensory neurons and the CNS in our model (*w*_ES_ = *w*_IS_ = 300) were also larger than the previous assumption (*w*_ES_ = *w*_IS_ = 1.95 in [10]). To sum, our mechanical model had larger viscosity and smaller muscular force whereas the circuit model had larger couplings between the CNS and their targets in the peripheral structures than the assumption in the previous theoretical work.

### The model reproduces previous observations in optogenetic experiments

**Fig. 6:**
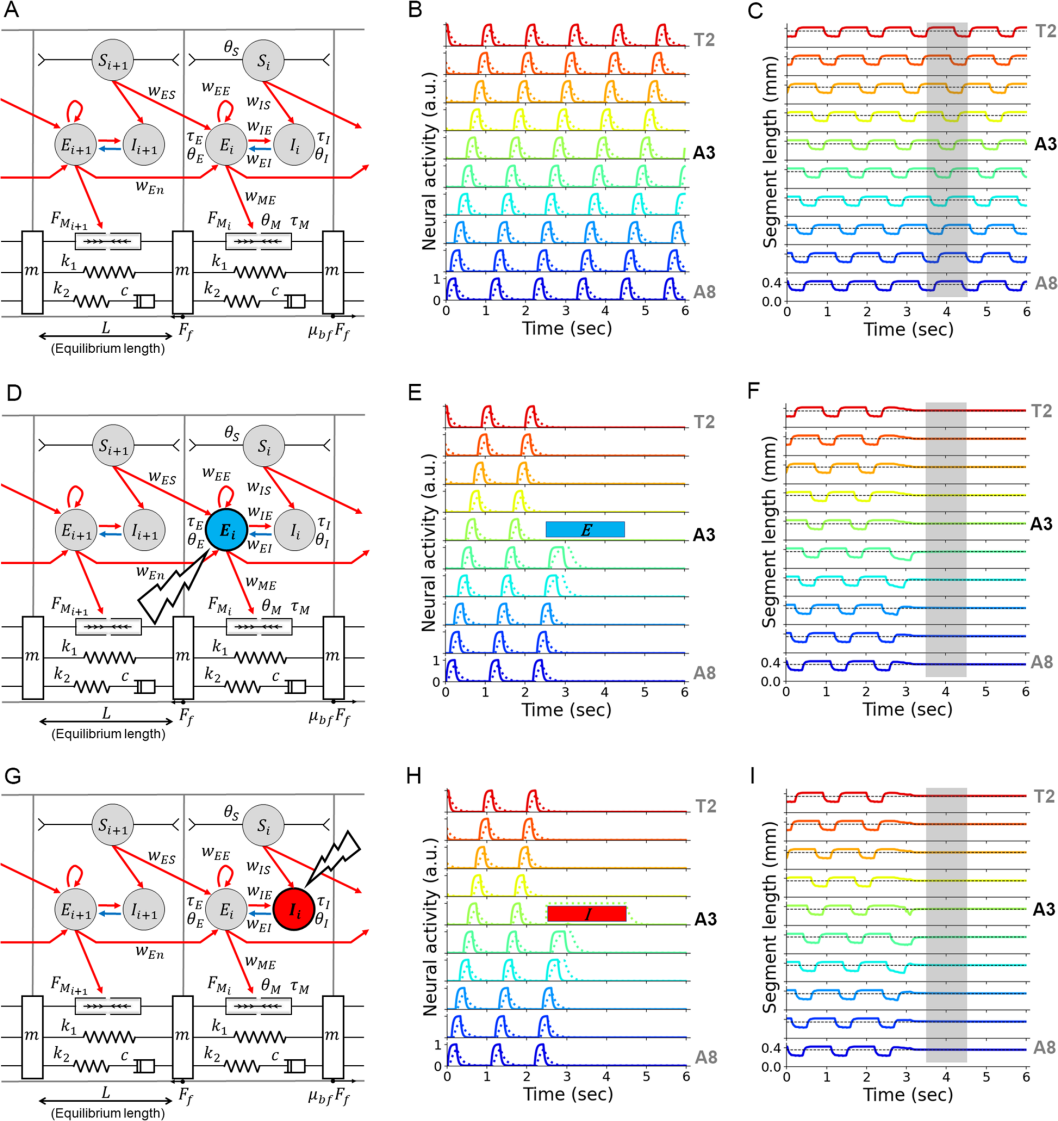
Reproduction of optogenetic experiments by the mathematical model. **A** A schematic of two segments in the mathematical models. **B** Activity of excitatory (solid lines) and inhibitory neurons (dotted lines) in A8–T2 segments by the model without optogenetic perturbation. **C** Traces of segment length in (**B**). Dotted lines denote the equilibrium length of the segments. A time period of 3.5–4.5 s is shaded to compare with (**F**) and (**I**). **D** A schematic of optogenetic silencing of excitatory neurons in a single segment shown by a blue disk. **E** Neural activity with the optogenetic silencing of excitatory neurons. Excitatory neurons in the A3 segment were silenced for 2 s, marked by a blue bar. Waves were arrested after the optogenetic silencing. **F** Traces of segment length in (**E**). Dotted lines denote the equilibrium length of the segments. In the later phase of the optogenetic silencing (the shaded region), the length of all the segments returned to the equilibrium length. **G** A schematic of optogenetic activation of inhibitory neurons in a single segment shown by a red disk. **H** Neural activity with the optogenetic activation of inhibitory neurons. Inhibitory neurons in the A3 segment were activated for 2 s, marked by a red bar. Waves were arrested after the optogenetic activation. **I** Traces of segment length in (**H**). Dotted lines denote the equilibrium length of the segments. In the later phase of the optogenetic activation of inhibitory neurons (the shaded region), the length of all the segments returned to the equilibrium length. Plot colours in **B**, **C**, **E**, **F**, **H**, and **I** correspond to those in Fig. 1F

To validate our model, we simulated two optogenetic perturbation studies reported previously and compared the results with the experimental observations [32, 53]. First, by optogenetic silencing of motor neurons in a few segments, propagation of contraction from the posterior to anterior segments was arrested, and all the segments were relaxed [53]. In the previous simulation study [10], although propagation was arrested by silencing the excitatory group in one segment, its neighbouring segment kept contracted instead of being relaxed. We silenced excitatory neurons in the A3 segment for 2 s in our neuromechanical model (Fig. 6A–F) and found that the silencing arrested the propagating wave (Fig. 6E). Furthermore, all the segments were relaxed by this optogenetic silencing (Fig. 6F), which is consistent with the experimental observation [53]. Inada et al. (2011) demonstrated that crawling was resumed after removing optogenetic silencing [53]. This phenomenon was not reproduced in our simulation: No wave was generated after optogenetic silencing (Fig. 6E, F), although waves were resumed after a short period of silencing (Additional file 7: Fig. S7). As pointed out in Pehlevan et al. [10], this would be due to the lack of such a mechanism in the neural circuit model. To sum, our model successfully reproduced the observation that the silencing excitatory neurons, particularly motor neurons, in a single segment arrested the propagation of peristaltic waves and relaxed all the segments.

Next, we tested the effect of activation of inhibitory neurons on crawling behaviour. A previous study reported that activation of inhibitory premotor interneurons PMSIs arrested wave propagation and caused the relaxation of the whole body [32]. Therefore, we activated inhibitory neurons in the A3 segment in the neuromechanical model (Fig. 6G). By this treatment, waves were arrested (Fig. 6H), and the whole segments returned to their equilibrium length (Fig. 6I). Accordingly, our neuromechanical model successfully reproduced the two optogenetics experiment results.

### Measurement of forward crawling behaviour in a low-friction condition and validation of the model

**Fig. 7:**
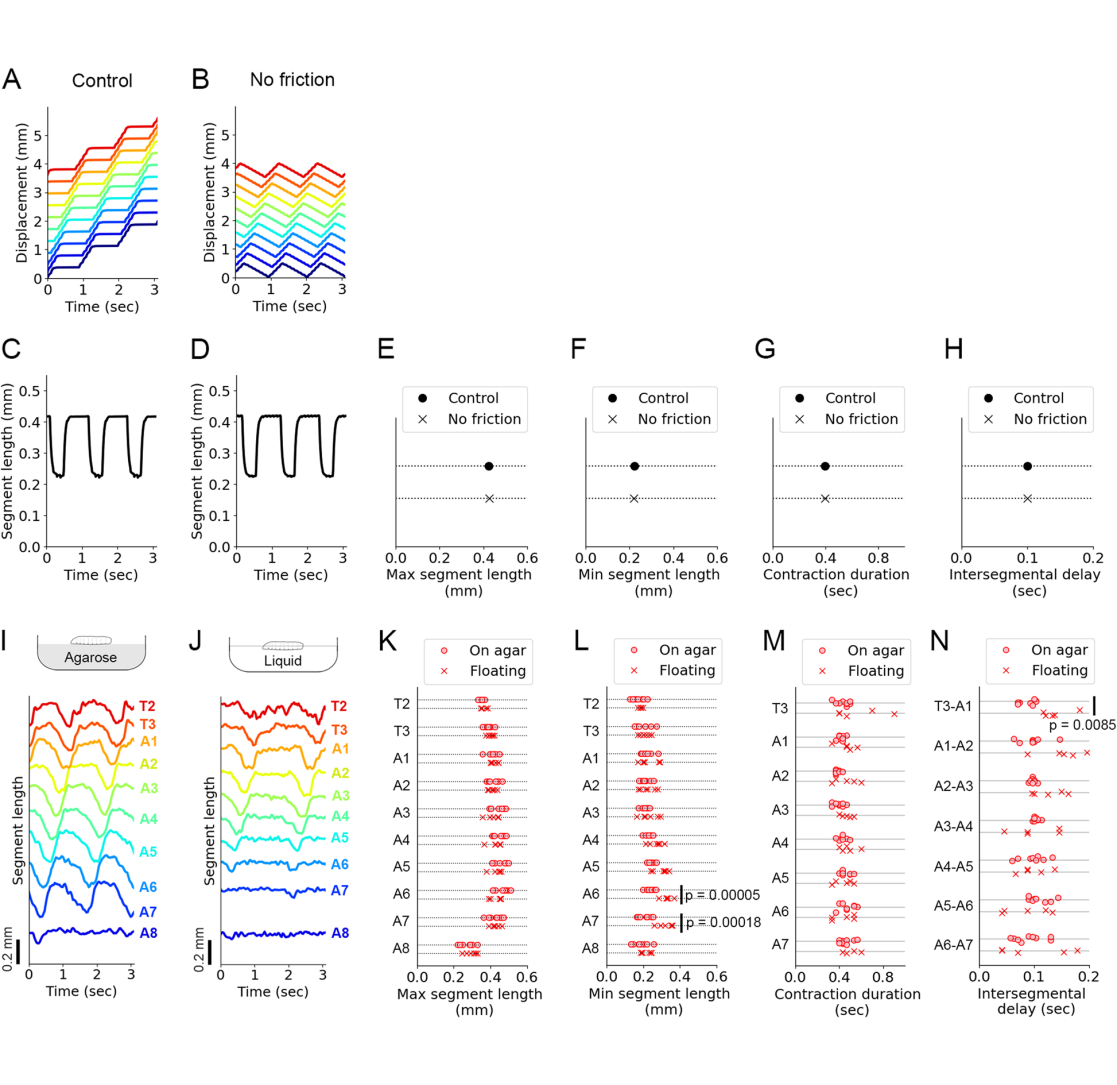
Mathematical simulation and experimental analysis of crawling behaviour in low friction conditions. **A**, **B** Simulation results of kymograph in the optimized condition (**A**) and the absence of friction (**B**). **C**, **D** Simulation results of segmental length in the optimized condition (**C**) and the absence of friction (**D**). **E**–**H** Comparison of four kinematic parameters in simulations between the optimized condition (control) and the absence of friction (no friction). **E** Maximum segment length. **F** Minimum segment length. **G** Contraction duration. **H** Intersegmental delay. **I**, **J** Plots of segment length measured by the experiments of larvae on agarose (**I**) and those floating in liquid (**J**). **K**–**N** Comparison of four kinematic parameters obtained by the experiments of larvae on agarose (on agarose) and larvae in a low friction condition (floating). The *p*-values show the result of Student’s *t*-test. **K** Maximum segment length. **L** Minimum segment length. **M** Contraction duration. **N** Intersegmental delay

Friction was a reaction force of a propelling force that drove larval movement. Although friction was necessary to move the centroid of larvae, it was unclear whether friction was required to generate the patterned segmental motion. In the previous theoretical study, two phenomena in segmental dynamics in a low-friction condition were simulated: Each segment contracted more, and intersegmental propagation delay became longer [10]. Our neuromechanical model tested the segmental dynamics in no friction conditions (Fig. 7A–H). The simulation results showed that the range of segment contraction did not change even in the no friction condition (Fig. 7C–F). Also, the duration of segmental contraction was not affected either (Fig. 7G). Furthermore, with respect to the wave propagation, the intersegmental delay was not changed in no friction condition (Fig. 7H). Accordingly, in our mathematical model, segmental dynamics was robust in the no friction condition.

To reveal the roles of friction on larval crawling, we measured the body segment kinematics in crawling larvae in a low-friction environment. We used a high concentration sugar solution (66% w/w sucrose) and floated third-instar larvae on it. To visualize the segment boundaries of larvae, we used *tubP-Gal4, UAS-fondue::GFP* fly larvae as described above (Fig. 1A). We compared the crawling motion between on the agarose substrate (Fig. 7I) and in the sugar solution (low friction environment) (Fig. 7J). Floating larvae could exhibit crawling behaviour (Fig. 7I), suggesting that the interaction with a substrate was not required to generate peristaltic motion. They could not move forward in the sugar solution, probably due to a lack of the propelling force that was the reaction force of friction or because of viscous resistance in the sugar solution. We also found that the number of waves was fewer in floating larvae than larvae on an agarose substrate (agarose 45.5 ± 3.5 crawls/min, *n* = 6 larvae; floating 6.8 ± 2.6 crawls/min, *n* = 6 larvae; *p* = 9.0 ×10^−6^, Welch’s *t*-test; Additional file 8: Fig. S8A). This observation implied that friction could be involved in the initiation of crawling. This phenomenon could not be observed in our neuromechanical model (Additional file 8: Fig. S8B) probably because our model did not include a mechanism to control the initiation of waves independent of the completion of waves. How each crawling wave was initiated and how the temporal gap between consecutive waves was regulated remained important open questions. Since our focus in this study was on the segmental and intersegmental dynamics, we analysed the kinematics of every single peristaltic wave.

First, we examined the segmental dynamics in the low friction condition and found that the range of segment contraction did not change in most of the segments in floating larvae (Fig. 7K, L). In addition, the contraction duration of each segment was not affected in the floating condition (Fig. 7M). These observations were consistent with our model results (Fig. 7E–G). In the posterior segments A6 and A7, however, there was a difference between larvae on agarose and floating larvae. The range of contraction at the posterior segments was reduced in floating larvae (Fig. 7L). Since forward crawling started at the posterior segments, this observation implied that friction would be crucial to initiate crawling. This implication was consistent with the observation of the reduction in crawling numbers in floating larvae (Additional file 8: Fig. S8A). Second, we analysed the intersegmental delay in the floating larvae. In most segments, the intersegmental delay was consistent between larvae on agarose and floating larvae (Fig. 7N). However, the intersegmental delay between T3 and A1 was increased in floating larvae. Since this phenomenon was observed in the limited anterior segment, it would be possible that other behaviour, including head-sweeping that occurs in the anterior segments, was affected in the low friction condition. To sum, the kinematics in larval crawling was almost similar between on the agarose substrate and in low-friction condition. This observation was consistent with the result of our neuromechanical model.

### Contribution of sensory feedback and CNS

**Fig. 8:**
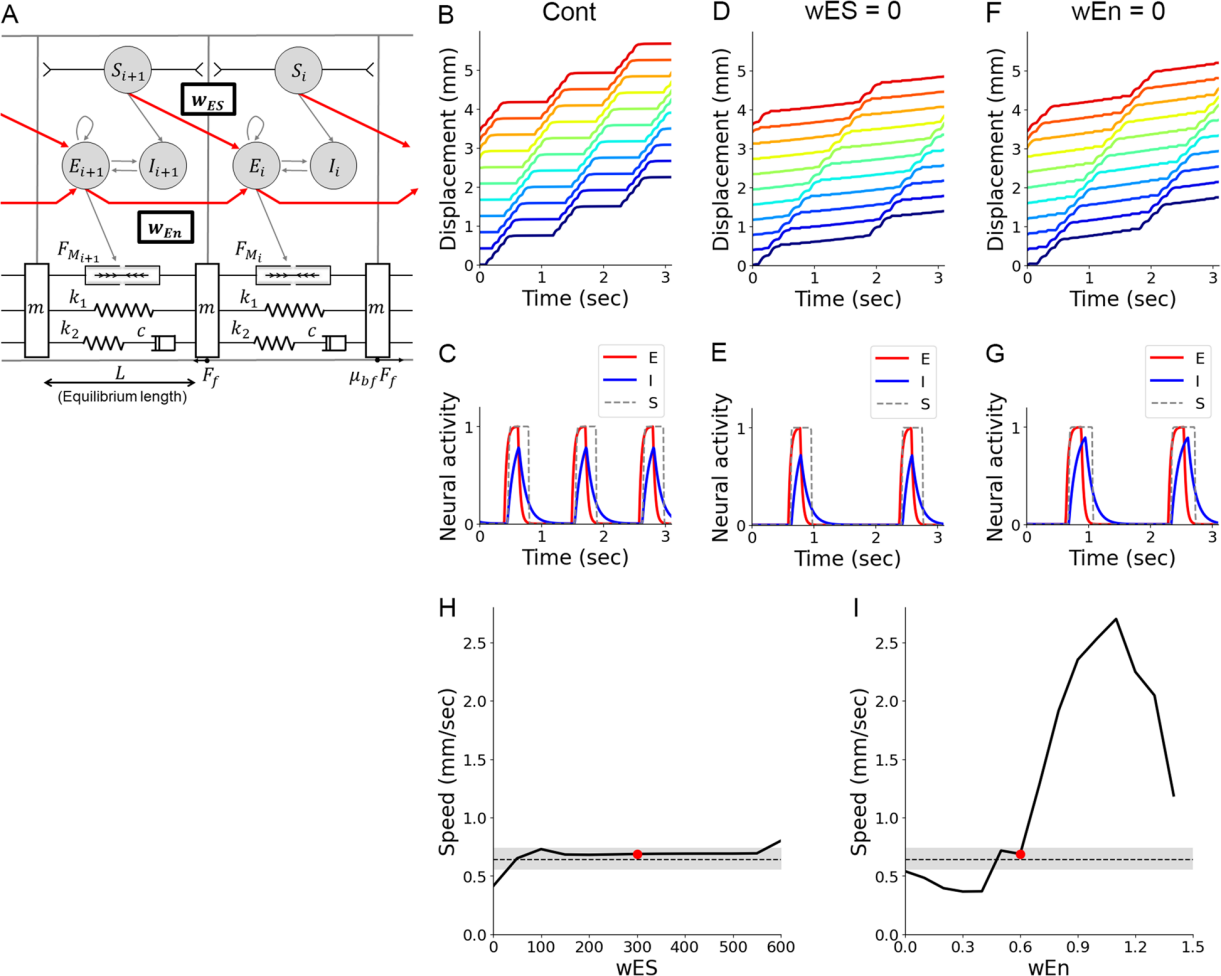
Sensory feedback and central connectivity are both involved in controlling crawling speed. **A** The weights of intersegmental proprioceptive sensory feedback *w*_ES_ and intersegmental coupling *w*_En_ in the neuromechanical model. **B**, **C** Simulation in the optimized condition. **B** Segmental boundary kymograph. **C** Activity of excitatory (red line), inhibitory (blue line), and sensory neurons (dotted lines) in a single segment. **D**, **E** Simulation in the absence of proprioceptive sensory feedback. **D** Segmental boundary kymograph. **E** Activity of excitatory (red line), inhibitory (blue line), and sensory neurons (dotted lines) in a single segment. **F**, **G** Simulation in the absence of intersegmental connections. **F** Segmental boundary kymograph. **G** Activity of excitatory (red line), inhibitory (blue line), and sensory neurons (dotted lines) in a single segment. **H**, **I** Plots of speed as proprioceptive sensory feedback *w*_ES_ (**H**) or intersegmental coupling *w*_En_ was perturbed (**I**). Grey-shaded regions show the range of speed observed in the experiment with third-instar larvae. Red dots indicate the optimized simulation condition

The CNS and proprioception were both involved in locomotion. The significant contribution of sensory feedback in larval crawling was shown experimentally [44]. This observation raised a hypothesis that proprioception could cause the propagation signal to the neighbouring segment through intersegmental sensory feedback [44]. A simulation based on this assumption showed that the model circuit with intersegmental feedback could normally generate propagation waves without an intersegmental connection in the CNS [10]. On the other hand, several key interneurons regulating larval locomotion had been identified [32, 54–56], suggesting that interneurons in the CNS should be involved in wave propagation. Aiming to reproduce the experimental observation of the involvement of the CNS in crawling, we tested the contribution of intersegmental sensory feedback and the intersegmental connection in the CNS to peristaltic motion by our neuromechanical model (Fig. 8). When the sensory feedback was silenced (*w*_ES_ = 0), the crawling speed was reduced (Fig. 8B, D, and H). This was consistent with the previous simulation result [10] and the experimental result [44]. When the intersegmental central connection was blocked (*w*_En_ = 0), the speed of crawling was decreased too (Fig. 8B, F, and I). This result was also consistent with observations that interneurons in the CNS were involved in crawling speed. While the speed was reduced in both cases, the neural mechanisms would be different. When sensory feedback was blocked, the activity of inhibitory neurons was attenuated (Fig. 8C, E). Meanwhile, when the intersegmental central connection was blocked, the activity of inhibitory neurons was enhanced (Fig. 8C, G). This observation suggested that sensory feedback and the CNS were involved in the speed control by distinct mechanisms. To examine their roles in detail, we perturbed *w*_ES_ and *w*_En_ from the optimal values. The speed of crawling was robust to the change of *w*_ES_ (Fig. 8H), whereas increase in *w*_En_ led to a drastic change in crawling speed (Fig. 8I). In particular, crawling speed depended on *w*_En_ nonlinearly with a peak. To sum, this simulation result was consistent with the experimental observations that interneurons in the CNS should be involved in the regulation of crawling behaviour.

**Fig. 9:**
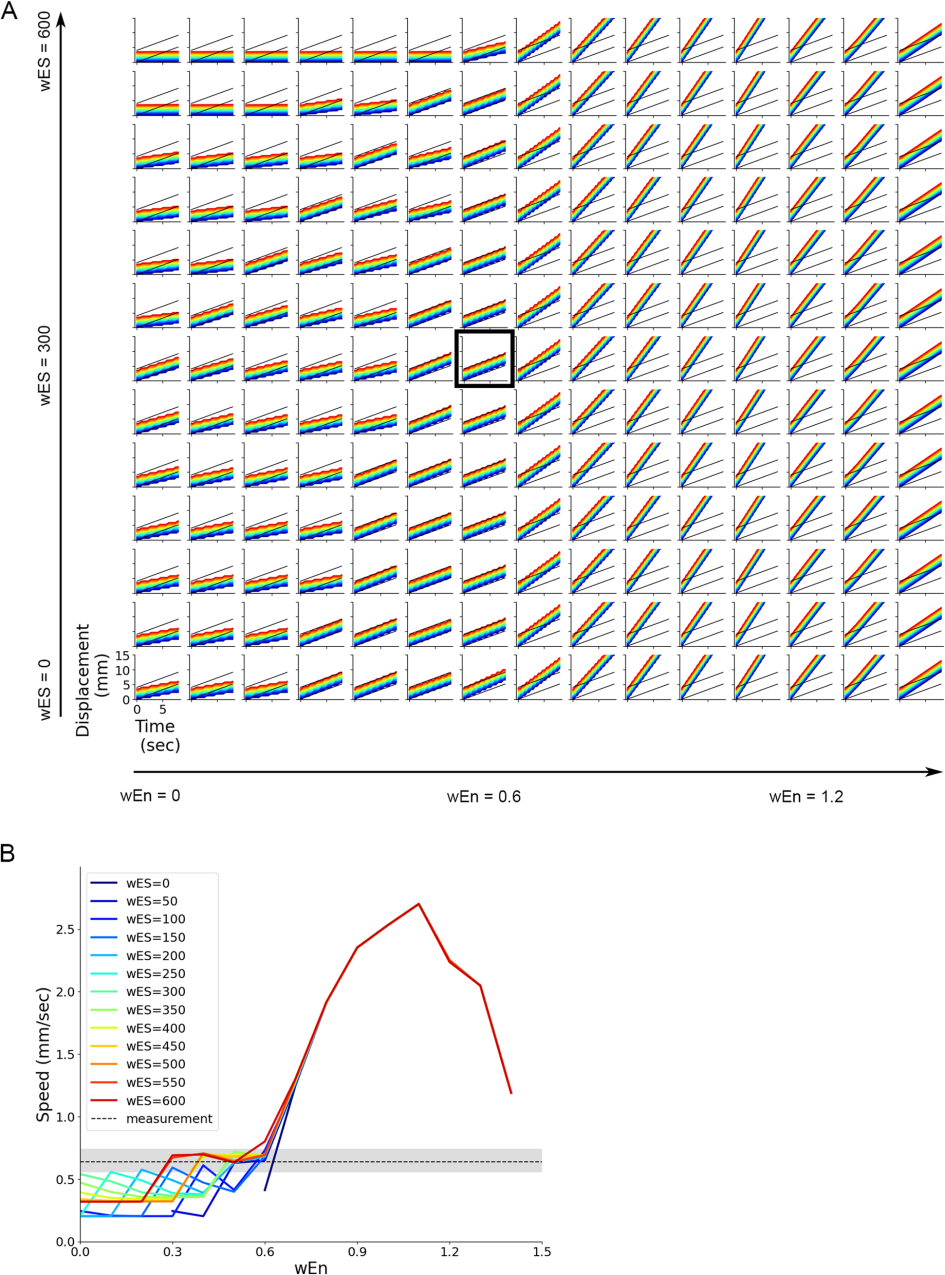
Interaction between *w*_ES_ and *w*_En_ in controlling crawling speed. **A** Plots of segment displacement in varied combinations of proprioceptive sensory feedback *w*_ES_ (along the vertical axis) and intersegmental coupling *w*_En_ (along the horizontal axis). The plot enclosed in a black rectangle corresponds to the optimized condition. **B** A plot of speed as both proprioceptive sensory feedback *w*_ES_ and intersegmental coupling *w*_En_ were perturbed. A grey line and a shaded region show the average and the range of speed observed in the experiment with third-instar larvae, respectively

Next, we analysed the interaction between intersegmental sensory feedback and intersegmental connection in the CNS. We perturbed *w*_En_ and *w*_ES_ simultaneously and plotted the displacement of larvae in a two-dimensional diagram (Fig. 9A). The plot showed that crawling could be realized in the wide area of this parameter space. When *w*_En_ was larger than the value in the optimized condition (0.6), *w*_En_ played a dominant role in speed control compared to *w*_ES_ (Fig. 9B). On the other hand, in the regime of smaller *w*_En_, both *w*_En_ and *w*_ES_ were involved in crawling speed. This result indicated the strength of central connectivity in the CNS should play a pivotal role in controlling the speed of crawling behaviour.

### Contribution of excitatory and inhibitory neurons in crawling

**Fig. 10:**
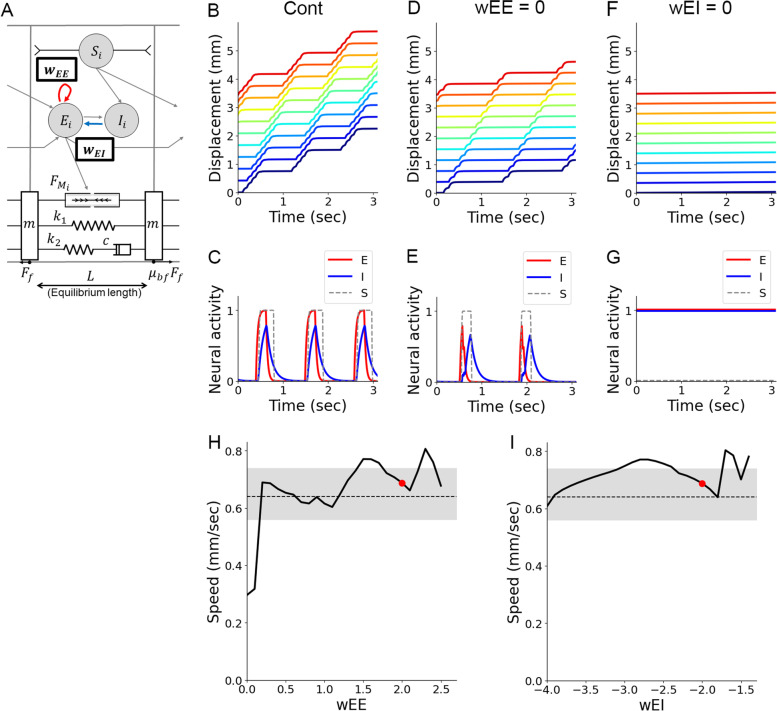
Intrasegmental excitatory and inhibitory connections are both involved in controlling crawling speed. **A** The weights of intrasegmental connections between excitatory neurons (*w*_EE_) and intrasegmental connections from inhibitory neurons to excitatory neurons (*w*_EI_) in the neuromechanical model. **B**, **C** Simulation in the optimized condition. **B** Segmental boundary kymograph. **C** Activity of excitatory (red line), inhibitory (blue line), and sensory neurons (dotted lines) in a single segment. **D**, **E** Simulation in the absence of intrasegmental connections between excitatory neurons. **D** Segmental boundary kymograph. **E** Activity of excitatory (red line), inhibitory (blue line), and sensory neurons (dotted lines) in a single segment. **F**, **G** Simulation in the absence of intrasegmental connections from inhibitory neurons to excitatory neurons. **F** Segmental boundary kymograph. **G** Activity of excitatory (red line), inhibitory (blue line), and sensory neurons (dotted lines) in a single segment. **H**, **I** Plots of speed as the weight of intrasegmental connections between excitatory neurons *w*_EE_ (**H**) or the weight of connections from inhibitory neurons to excitatory neurons *w*_EI_ was perturbed (**I**). Grey-shaded regions show the range of speed observed in the experiment with third-instar larvae. Red dots indicate the optimized simulation condition

Based on the observation suggesting a significant contribution of the CNS to crawling behaviour, we next examined the involvement of excitatory and inhibitory neurons in larval locomotion. To this aim, we perturbed intrasegmental connection weight: *w*_EE_ for excitatory connections and *w*_EI_ for inhibitory connections (Fig. 10A–G). Crawling speed decreased when blocked excitatory connections (Fig. 10B, D). Since the traces of excitatory and inhibitory neurons were different from those in blocking intersegmental excitatory connections (Figs. 8E and 10E), the speed reduction mechanism should be different between silencing the intrasegmental excitatory connections and intersegmental excitatory connections. On the other hand, when the activity of inhibitory neurons was suppressed, larvae could not exhibit crawling (Fig. 10F). In this case, all the neurons exhibited tonic hyperactivity due to a lack of inhibition (Fig. 10G). To further reveal the involvement of *w*_EE_ and *w*_EI_, we perturbed them in the range where larvae could exhibit crawling. The plots showed that speed was related to *w*_EE_ and *w*_EI_ in non-monotonical manners (Fig. 10H, I). This observation indicated that both excitatory and inhibitory neurons controlled crawling speed.

**Fig. 11:**
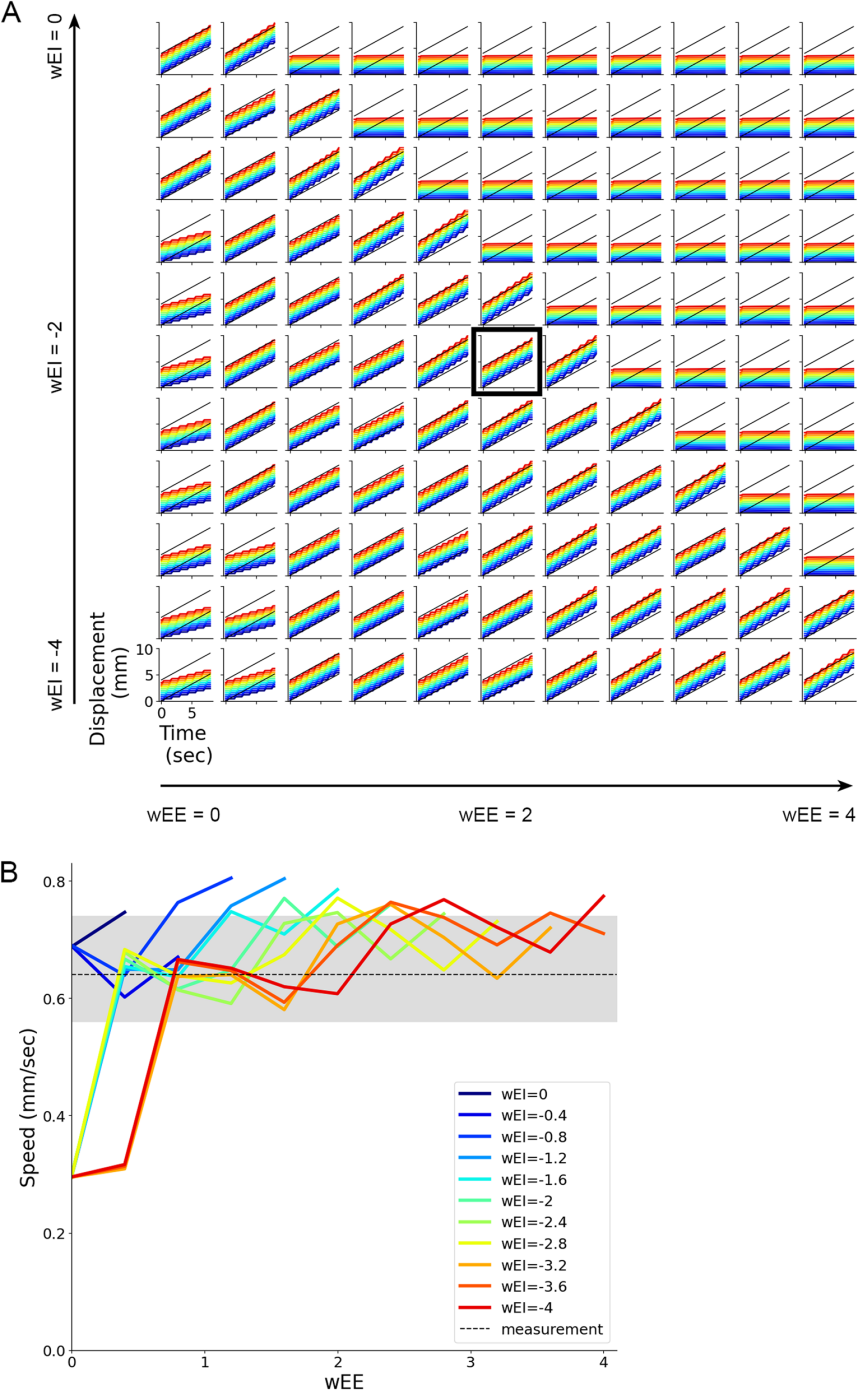
Interaction between w_EE_ and w_EI_ in controlling crawling speed. **A** Plots of segment displacement in varied combinations of the weights of intrasegmental connections between excitatory neurons *w*_EE_ (along the horizontal axis) and from inhibitory neurons to excitatory neurons *w*_EI_ (along the vertical axis). The plot enclosed in a black rectangle corresponds to the optimized condition. **B** A plot of speed as both *w*_EE_ and *w*_EI_ were perturbed. A grey line and a shaded region show the average and the range of speed observed in the experiment with third-instar larvae, respectively

Next, we analysed the interaction between excitatory and inhibitory neurons. Displacement of larvae with different values of *w*_EE_ and *w*_EI_ was plotted two-dimensionally. The diagram indicated that when *w*_EE_ was large and *w*_EI_ was close to zero (the upper right side of the diagram), larvae could not generate crawling (Fig. 11A). On the other hand, when *w*_EE_ was small (weak excitatory connection) or *w*_EI_ was small (strong inhibitory connection) that corresponded to the lower-left region of the diagram, and crawling speed was reduced. Interestingly, as long as *w*_EE_ and *w*_EI_ were balanced (the diagonal region of the diagram), crawling speed was consistent. The deviation in speed when *w*_EE_ and *w*_EI_ were perturbed was within the range of variability observed experimentally (Fig. 11B). These results indicated two points: The balance between excitatory and inhibitory connections within segments was critical to generating peristaltic motion, and, as long as they were balanced, crawling speed would not change by perturbation in intrasegmental connection weights.

### Prediction in the neural network in sister species of *Drosophila melanogaster*

**Fig. 12:**
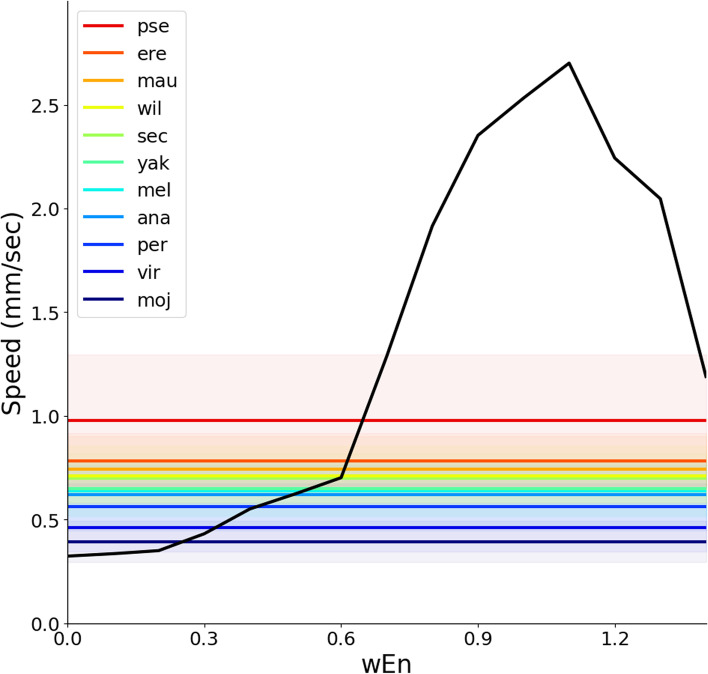
Prediction of the strength of intersegmental connections in sister species in genus *Drosophila*. The black curve shows the relation between the intersegmental coupling *w*_En_ and crawling speed obtained by the neuromechanical model based on the measurement of *Drosophila melanogaster* larvae. This plot was obtained by averaging out the weight of proprioceptive sensory feedback *w*_ES_ in Fig. 9B. The average and standard error mean of speed in species in the genus *Drosophila* are shown by solid lines and shaded areas, respectively. The speed data was calculated from the data in [57]. The 11 *Drosophila* (*D.*, hereafter) species consisted of *D. ananassae* (ana), *D. erecta* (ere), *D. mauritiana* (mau), *D. melanogaster* (mel), *D. mojavensis* (moj), *D. persimilis* (per), *D. pseudoobscula* (pse), *D. sechellia* (sec), *D. virilis* (vir), *D. willistoni* (wil), and *D. yakuba* (yak)

A comparison between the contribution of intersegmental (Fig. 9B) and intrasegmental (Fig. 11B) connections to crawling speed suggested that intersegmental connections in the CNS had dominant roles in regulating crawling speed. We noticed that this observation could allow us to predict neural circuits for fly larval crawling. A recent study reported an inter-specific variation in crawling speed among eleven species in the genus *Drosophila* [57]. This study showed that some sister species crawled faster than *Drosophila melanogaster*, and the others locomoted slower. We attempted to make a prediction about the intersegmental connection in the CNS of these species based on their crawling speed. To predict the property of neural circuits in these sister species, we overlaid the speed data of the species on the graph showing speed dependency on *w*_En_ (Fig. 12). To compensate the difference in experimental conditions between this study and Matsuo et al. [57], the speed of each species was normalized by the speed of *Drosophila melanogaster*. To analyse the dependency on *w*_En_, the dependency of *w*_ES_ was averaged out from Fig. 9B. The result showed that all of the eleven horizontal lines demonstrating the crawling speed of sister species crossed to the speed-*w*_En_ relation curve (Fig. 12). This plot suggested that some species showing faster crawling, *Drosophila pseudoobscura* for example, should have strong intersegmental connections in the CNS while those crawling slower such as *Drosophila mojavensis*, should have weaker intersegmental central connections. This prediction would be testable by interspecific comparison based on the anatomy of their central nervous system or physiological recording.

## Discussion

In this work, we built a neuromechanical model based on the physical properties of fly larvae. The model successfully described crawling behaviour quantitatively. First, we quantified segmental dynamics in larval crawling and obtained seven parameters that characterize the kinematics of peristalsis (Figs. 1 and 2). Then, using a tensile tester, viscoelasticity and muscular force of larvae were measured (Figs. 3 and 4). These results suggest that the larval body could be modelled as a chain of mass and SLS modules (Fig. 4), distinct from the previous modelling for larvae [10, 39, 41]. By incorporating the material properties and optimizing the parameters in the neural circuit, our model succeeded in reproducing larval crawling quantitatively (Fig. 5). Furthermore, this model could describe the observation in optogenetic studies (Fig. 6) and crawling in a low-friction condition tested in this study (Fig. 7). Perturbation analyses indicated the importance of intersegmental connections in the CNS, contrasting with previous studies (Figs. 8 and 9). Based on this observation, we predicted the intersegmental connections in the CNS in sister species in the genus *Drosophila* (Fig. 12).

One striking finding from our model contrasts with previous studies: the significant contribution of intersegmental connections in the CNS to crawling speed. Although several interneurons involved in speed control have been reported [32, 54–56], previous simulation studies were not consistent with these observations. Our model had larger viscosity and smaller contraction forces (Additional file 3: Fig. S3) than previous parameter regimes, implying that each segment is hard to move. The large weight of neuromuscular connection (*w*_ME_) and that of sensory feedback (*w*_ES_) compensated for this difficulty (Additional file 3: Fig. S3). With the strong interaction between the CNS and peripheral organs (muscles and proprioceptors), the CNS could play a significant role in controlling crawling speed (Fig. 8I). This finding demonstrates the significance of intersegmental connection in the CNS. It also highlights the importance of mechanical properties in the larval body to understand the dynamics of larval locomotion.

We measured the viscoelastic properties of larvae by the stress-relaxation test. It should be noted that there were some limitations in this measurement. First, we obtained these passive properties of the body by measuring unanaesthetized larvae. We analysed the data without any spontaneous contraction in the stress-relaxation test. However, there remained a possibility that infinitesimal muscular tension was induced by proprioceptive feedback upon stretching. By blocking action potentials in motor neurons while keeping spontaneous vesicle release, it would be able to suppress feedback from proprioception to measure purer viscoelasticity. Second, we approximated the viscoelasticity for the contraction in the range of 0.2 mm, which larvae showed in crawling, by the viscoelasticity for the extension in the range of 0.04 mm, which we adopted in the stress-relaxation test. In real larval crawling, asymmetricity between extension and contraction and nonlinearity in the viscosity would affect the kinematics. By incorporating these factors, it would be able to reproduce the kinematics more quantitatively.

The model in this study could describe forward crawling, the most frequent behaviour in larvae. Recent neuroscience studies have revealed numerous neural circuit modules involved in distinct aspects of behaviour, including speed control [32], bilateral coordination [35], intrasegmental coordination [36], intersegmental coordination [31], backward crawling [58, 59], turning [60], escaping [61], and sensory input-guided navigation [34, 62]. By integrating these circuit modules, it would be possible to establish a neuromechanical model that reproduces multiple and natural larval behaviours. Furthermore, the neuromechanical model in this study can potentially serve biomimetics. In recent decades, more and more soft robots, endowed with new capabilities relative to the traditional hard ones, have been designed to exhibit complex movements [63–66]. Taking *Drosophila* larvae as a prototype, bionic structures can be established with high dexterity to explore the unstructured environments. By harnessing perturbation analyses on the neuromechanical model, the locomotion ability of the soft larval robots could be tuned and optimized for required applications.

## Conclusions

We established a neuromechanical model based on physical measurements of fly larvae to describe larval crawling motion. The model succeeded in quantitatively reproducing the segmental dynamics of larval locomotion measured experimentally in this work. The model also reproduced previous optogenetic observations. In addition, this model predicted a regular peristaltic motion in a low-friction condition, and this property was confirmed experimentally in this study. Furthermore, the model predicted an essential contribution of intersegmental neural connections in the central nervous system, contrasting with a previous report. This hypothesis provided a testable prediction for the diversity in intersegmental connectivity in sister species in the genus *Drosophila*. This study provided a new foundation to investigate crawling kinematics in soft-bodied animals and soft robot engineering.

## Methods

### Fly strains

We used the third-instar larvae in all the experiments. To quantify segment dynamics, we used *tubP-Gal4, UAS-fondue::GFP* (Bloomington #5138, #43646), which labels the segment boundaries [45]. In the viscoelasticity measurements and the spontaneous contraction force measurement, we used a wild-type strain *Canton S*. For the optogenetic experiment, *OK6-Gal4* [67] and *UAS-ChR2[T159C]::YFP* [31] were used.

### Image acquisition and segment boundary annotation

Third-instar larvae of *tubP-Gal4, UAS-fondue::GFP* were gently washed in deionized water to remove residual food from the body surface. Then, individual larvae were placed on a flat agarose stage (1.5% agarose). Behavioural videos were captured by a charge-coupled device (CCD) camera (XCD60, Sony, Japan) mounted on an Olympus stereomicroscope (SZX16, Olympus, Japan). Images were acquired at 30 Hz. We used the Fiji software [68] to manually annotate the right and left ends of every segment boundary (from the anterior boundary of the T2 segment to the posterior boundary of the A8 segment; see Fig. 1A, *n* = 9 larvae, biological replicates). The midpoint of the right and left ends of a boundary was used as the position of the boundary.

### Spontaneous contraction force and viscoelasticity measurement

Third-instar larvae in the feeding stage were washed with deionized water and dried with paper. Insect pins were inserted into the head and tail. The pin in the head was bent to form a loop to hook to a paper clip that was hung on the hook of the tensile sensing machine, while the pin in the tail was used to fix the body on the PDMS (Polydimethylsiloxane, Sylgard 184, Toray, Japan) silicone block, held by the tong on the SHIMADZU EZ-S platform with a 5-N load cell. The experimental equipment is shown in Fig. 3A (for the measurement of spontaneous contraction force) and Fig. 4A (for the stress relaxation test). The larval body was kept on the vertical axis for measurement. The baseline of force was calibrated by values measured before applying external elongational force. All the experimental procedures were performed at room temperature.

In the measurement of the spontaneous contraction force, we recorded eleven contraction events from three larvae that are biological replicates (Fig. 3E).

During the stress relaxation test to measure viscoelasticity, we applied a constant strain of 0.4 mm, about 10% of the body length (3.53 ± 0.12 mm, *n* = 9 larvae, biological replicates), to the larvae (*n* = 6 larvae, biological replicates). Then, the stress decreased until it reached a plateau after a while, as shown in Fig. 4H. To fit the stress relaxation curve to mechanical analogues, we adopted the Maxwell, Kelvin-Voigt, and standard linear solid (SLS) models. These models give the relationship between joint force *F* and displacement ∆*L*. Detailed functions are described as follows:

$$\frac{F}{c}+\frac{1}{k}\frac{dF}{dt}=\frac{d\Delta L}{dt}\kern0.75em \mathrm{Maxwell}\ \mathrm{model}$$

$$F=k\Delta L+c\frac{d\Delta L}{dt}\kern0.75em \mathrm{Kelvin}-\mathrm{Voigt}\ \mathrm{model}$$

$$F+\frac{c}{k_2}\frac{dF}{dt}={k}_1\left(\Delta L+c\frac{k_1+{k}_2}{k_1{k}_2}\frac{d\Delta L}{dt}\right)\kern0.75em \mathrm{SLS}\ \mathrm{model}$$

where *k* (or *k*_1_and *k*_2_ in the SLS model) and *c* are the elastic and damping constants, respectively, and *t* is time. The total displacement of 0.4 mm was realized by pulling the larval body at 1 mm/min. During the stress relaxation experiments, this elongation time (24 s) is much shorter than that for relaxation (576 s), and thus, the displacement is regarded as the step function *L*(*t*) = *L*_0_*H*(*t* − *t*_0_) and initial force is *F*(0) = 0*N*, where *L*_0_ = 0.4 mm, and *H*(*t*) is the Heaviside step function. In this case, the corresponding stress relaxation functions in these models are described as follows:

$$F=k{e}^{-\frac{k}{c}\left(t-{t}_0\right)}{L}_0H\left(t-{t}_0\right)\kern0.5em \mathrm{Maxwell}\ \mathrm{model}$$

$$F=k{L}_0H\left(t-{t}_0\right)+c{L}_0\delta \left(t-{t}_0\right)\kern0.5em \mathrm{Kelvin}-\mathrm{Voigt}\ \mathrm{model}$$

$$F(t)=\left[{k}_1+{k}_2{e}^{-\frac{k_2\left(t-{t}_0\right)}{c}}\right]{L}_0H\left(t-{t}_0\right)\kern0.5em \mathrm{SLS}\ \mathrm{model}$$

where *t*_0_ ≥ 0. By fitting these curves to the stress-relaxation measurement data, we obtained the spring constants and damping coefficients. We used Python 3.7 for the curve fitting.

### Contraction force measurement with optogenetics

For optogenetic activation of motor neurons, we used the *OK6-GAL4, UAS-ChR2* line [32]. The early third-instar larvae were selected and put into ATR (all-trans-retinal) containing yeast paste, the concentration of which was 1 mM. These larvae were reared at 25 °C in the dark for 1 day [69]. Afterwards, the third-instar larvae were prepared to measure tension force as described above (the “Spontaneous contraction force and viscoelasticity measurement” section). Blue light-emitting diode (LED) light (455 nm, 5.7 nW/mm^2^, M455L3, ThorLab) was used to stimulate Channelrhodopsin2 (ChR2) expressed in motor neurons, which leads to the contraction of the larval body. In each stimulation, the blue light was applied for 2 s, and the evoked force was monitored. Seven larvae (biological replicates) were used in the measurement, and each measurement took two to 5 min. The optogenetically induced forces were measured by the differences between forces during illumination and no illumination (Fig. 3).

### Larval crawling in a low-friction environment

We used a high concentration sugar solution (66% w/w sucrose) and floated third-instar larvae in it. The genotype we used was *tubP-Gal4, UAS-fondue::GFP*, which allowed us to mark the segmental boundary from T2 to A8 (Fig. 1A, B). The locomotion (*n* = 6 larvae, biological replicates) was recorded via an SZX16 fluorescent microscope (Olympus, Japan) with a × 1.25 object lens at 30 frames/s, and its trajectory was measured by Fiji [68].

### Modelling

#### Body-substrate mechanics and modelling

The larval crawling stride consists of the piston and wave phases [26]. The piston phase constrains the larval body length to be almost constant, while the wave phase generates the propagation wave repetitively. To make it simple, we modelled the whole body of larvae as a chain of eleven segments.

We assumed that the segments in the model larva are entirely repetitive based on the observation in Figs. 1 and 2. Then, the body was modelled as a chain of the SLS units in series (Additional file 1: Fig. S1). The muscle groups were modelled as the tension actuator to accept efferent control from the neural circuit (Fig. 5B). ${F}_{M_i}$ is the muscular tension force in the *i*th segment to counteract the effect of body viscoelasticity and friction. The mechanics are described based on Newton’s second law as follows.

$$\left\{\begin{array}{c}m{\ddot{y}}_{-1}=m{\ddot{y}}_{10}={F}_{10}-{F}_0+{F}_{M_{10}}-{F}_{M_0}-{f}_{-1}\kern7em \\ {}m{\ddot{y}}_i={F}_i-{F}_{i+1}+{F}_{M_i}-{F}_{M_{i+1}}-{f}_i\kern1.5em i=0,\dots, n-2\kern3.25em \\ {}{y}_{-1}={y}_{10}+ nL\kern21em \end{array}\right.$$

$${F}_i+\frac{c}{k_2}\frac{d{F}_i}{dt}={k}_1\left({y}_{i-1}-{y}_i-L+c\frac{k_1+{k}_2}{k_1{k}_2}\left(\frac{d{y}_{i-1}}{dt}-\frac{d{y}_i}{dt}\right)\right)$$

$${\tau}_M{\dot{F}}_{M_i}=-{F}_{M_i}+{F}_{M_{\mathrm{max}}}{\sigma}_M\left({w}_{\mathrm{ME}}{E}_i-{\theta}_M\right)\kern2em i=0,\dots, n$$

$${\sigma}_M(v)=\frac{1}{1+{e}^{-{k}_Mv}}$$

where *y*_*i*_ is the segmental boundary position (Fig. 1B and Additional file 1: Fig. S1), *m* is the mass, *F*_*i*_ is the viscoelastic force, ${F}_{M_i}$ is the muscular tension force, *f*_*i*_ is the friction force (see below), *τ*_*M*_ is the time relaxation constant for the muscular tension, *w*_ME_ is the connection weight from the excitatory unit to the muscular actuator, *θ*_*M*_ is the threshold for the muscular tension, *k*_*M*_ is the gain in function *σ*_*M*_(*v*), and *n* is the total number of segments, equal to 11. *y*_−1_ is the position of the head, and *y*_10_ is the position of the tail. To model the piston phase, where the head and tail move concurrently, the posterior and anterior ends share the same velocity during crawling, as ${\dot{y}}_{-1}={\dot{y}}_{10}$ which is inferred from the third equation above. Each mass is subject to a joint force of the force from SLS modules, the muscular tension, and the friction force. The force from the SLS unit is affected by the segmental displacement, and the muscular tension force is regulated by the sigmoid function with gain *k*_*M*_.

As for the friction force, it exists during the interaction of the mechanical body with the substrate. We modelled that the joint force on a mass was zero when the speed of the mass equals zero, and the sum of the force from the SLS module and the muscular force was weak by giving the counterforce by the friction. In other cases, The friction was *F*_*f*_ in forward movement and *μ*_*bf*_*F*_*f*_ in backward movement. The ratio of forward to backward friction (*μ*_*bf*_) was introduced to the model as directional asymmetric, considering the anterior-posterior polarity in denticle bands. Since the backward friction is larger than the forward friction, the ratio is more than one. Accordingly, the friction on the mass was represented as:

$${f}_i=\left\{\begin{array}{c}{F}_{i,\operatorname{ext}}\kern12em {\dot{y}}_i=0\ \mathrm{and}-{\mu}_{bf}{F}_f\le {F}_{i,\operatorname{ext}}\le {F}_f\\ {}{F}_f\left({\sigma}_f\left({\dot{y}}_i\right)-{\mu}_{bf}{\sigma}_f\left(-{\dot{y}}_i\right)\right)\kern2.5em \mathrm{otherwise}\kern9.25em \end{array}\right.\kern1.25em i=-1,0,\dots, n-1$$

$${\sigma}_f(v)=\frac{1}{1+{e}^{-{k}_fv}}$$

where *f*_*i*_, *F*_*f*_, *μ*_*bf*_ (*μ*_*bf*_ > 1) individually represent the friction, forward friction, and ratio for forward-backward friction, respectively, and *F*_*i*, ext_ refers to the joint force of the force from the SLS unit and the muscular tension. As described above, when the mass is still and *F*_*i*, ext_ does not exceed the range of forward-backward friction, the friction force *f*_*i*_ should be equal to *F*_*i*, ext_ to cancel out the total force (the joint force) on the mass. Otherwise, the friction is either *F*_*f*_ or *μ*_*bf*_*F*_*f*_ depending on the movement direction.

#### Neuromuscular dynamics and modelling

The framework for the neural circuit is depicted in Fig. 5B. The neural circuit model is based on the model in Pehlevan et al. [10]. Neural dynamics is realized by the activities of excitatory and inhibitory populations of neurons in each segment under the Wilson-Cowan model [70, 71]. In *Drosophila* larvae, most proprioceptive neurons were active when the segment was contracted [43]. Referring to the model in Pehlevan et al. [10], the feedback from the sensory receptor in our model provides signals for the inhibitory neuron within the same segment and the excitatory neuron in the anterior segment. In this case, it can promote both local relaxation and forward propagation waves. This connectivity is consistent with the “mission-accomplished” model [44].

Under these assumptions, the neural dynamics of neural circuits were described as follows:

$$\left\{\begin{array}{c}{\tau}_E{\dot{E}}_i=-{E}_i+{\sigma}_E\left({w}_{\mathrm{EI}}{I}_i+{w}_{\mathrm{EE}}{E}_i+{w}_{\mathrm{En}}{E}_{i+1}+{w}_{\mathrm{ES}}{S}_{i+1}-{\theta}_E\right)\kern1.75em i=0,\dots, n-2\kern0.75em \\ {}{\tau}_I{\dot{I}}_i=-{I}_i+{\sigma}_I\left({w}_{\mathrm{IE}}{E}_i+{w}_{\mathrm{IS}}{S}_i-{\theta}_I\right)\kern13.5em i=0,\dots, n-2\kern0.75em \\ {}{\tau}_E{\dot{E}}_{10}=-{E}_n+{\sigma}_E\left({w}_{\mathrm{EI}}{I}_{10}+{w}_{\mathrm{EE}}{E}_{10}+{w}_{\mathrm{En}}{E}_0+{w}_{\mathrm{ES}}{S}_0+\mathrm{ini}-{\theta}_E\right)\kern6.5em \\ {}{\tau}_I\dot{I_{10}}=-{I}_{10}+{\sigma}_I\left({w}_{\mathrm{IE}}{E}_{10}+{w}_{\mathrm{IS}}{S}_{10}-{\theta}_I\right)\kern18.75em \end{array}\right.$$

$${S}_i={\sigma}_S\left(L{\theta}_S-{y}_{i-1}+{y}_i\right)\kern13em i=0,\dots, n-1$$

$${\sigma}_E(v)=\frac{1}{1+{e}^{-{k}_Ev}},\kern2em {\sigma}_I(v)=\frac{1}{1+{e}^{-{k}_Iv}},\kern2em {\sigma}_S(v)=\frac{1}{1+{e}^{-{k}_Sv}}$$

where *E*_*i*_ and *I*_*i*_ are the mean firing rates of the excitatory and inhibitory population in the *i*th segment, respectively; *τ*_*E*_ and *τ*_*I*_ are the relaxation time constant of the excitatory and inhibitory units, respectively; *w*_ab_ is their synaptic connection weight from population *b* to population *a*; *θ*_*E*_ and *θ*_*I*_ are the activation threshold of the excitatory and inhibitory units, respectively; *k*_*E*_, *k*_*I*_, and *k*_*S*_ are the sigmoid gain of excitatory, inhibitory, and sensory units, respectively; and *S*_*i*_ is the feedback strength from the sensory receptor in the *i*th segment. The circuit includes excitatory connections via *w*_IE_ and *w*_ME_ and inhibitory connections via *w*_EI_. When the segmental length becomes smaller than the threshold *Lθ*_*S*_, the sensory unit starts to be activated. To trigger the initial crawling, we introduced an external stimulus ini, a rectangular pulse (for 5 ms), on the excitatory population in the posterior terminal.

The values of the parameters are listed in Additional file 3: Fig. S3. All the simulation work is performed using stiff solver ode15s in MATLAB R2021a.

## Supplementary Information

**Additional file 1: Fig. S1.** A physical model with eleven segments. Based on the estimation of the number of segments, the fly larva was modelled by eleven segments. We assumed that *y*_10_ and *y*_−1_ were physically coupled. In the simulation results in this paper, segmental boundaries from *y*_10_ to *y*_0_ or segments from A8 to T2 were shown.

**Additional file 2: Fig. S2.** Fitting stress-relaxation test data by three viscoelastic models. The same stress-relaxation test data was fitted with the SLS model (A), Maxwell model (B), and Kelvin-Voigt model (C).

**Additional file 3: Fig. S3.** Our physical model for larval crawling and its parameters. (A) Schematic of the physical model for larval crawling. (B) List of parameters whose values were obtained by measurement using fly larvae. (C) List of parameters whose values were obtained by fitting to reproduce larval crawling by the simulation.

**Additional file 4: Fig. S4.** Perturbation analysis on viscoelasticity. Plots of speed as viscoelasticity was perturbed. (A) Perturbation of the spring constant *k*_1_. (B) Perturbation of the spring constant *k*_2_. (C) Perturbation of the dumping coefficient *c*. The horizontal axes were normalized by the optimized values (*k*_1_ _ *optimal*, *k*_2_ _ *optimal*, and *c* _ *optimal*), respectively. Grey shaded regions show the range of speed observed in the experiment with third-instar larvae. Red dots indicate the optimized simulation condition. The minimum value in the horizontal axis in the plot of (C) equals 1/400, which corresponds to the value in Pehlevan *et al.* (2016) [10].

**Additional file 5: Fig. S5.** Perturbation analysis on the asymmetricity in friction. Plots of speed as the friction asymmetricity was perturbed. The grey shaded region shows the range of speed observed in the experiment with third-instar larvae. The red dot indicates the optimized simulation condition.

**Additional file 6: Fig. S6.** Perturbation analysis on the maximum muscular force. Muscular force and segment displacement when the maximum muscular contraction force was perturbed. (A-D) Muscular forces in all the segments. Line colours correspond to those in Fig. 1B and C. (E-H) Displacement of the segmental boundaries. Line colours correspond to those in Fig. 1E and F. The ratio of the maximum contraction force to the optimized force: A and E, 100%; B and F, 75%; C and G, 50%; D and H, 25%.

**Additional file 7: Fig. S7.** Different durations of optogenetic stimulation in the simulation exhibit distinct phenotypes in the propagation of waves. (A) Schematic of optogenetic silencing of excitatory neurons in a single segment shown by a blue disk. (B) Neural activity with the optogenetic silencing of excitatory neurons. Excitatory neurons in the A3 segment were silenced for 0.5 seconds, marked by a blue bar. Waves were resumed just after the optogenetic silencing was removed. (C) Traces of segment length in (B). The dotted box shows the segment length of the posterior neighbouring segment during optogenetic stimulation. The arrow indicates that the neighbouring segment was contracted just after the optogenetic stimulus was removed. (D) Neural activity with the optogenetic silencing of excitatory neurons for 0.7 seconds. Excitatory neurons in the A3 segment were silenced as marked by a blue bar. Waves were arrested even after the optogenetic silencing was removed. (E) Traces of segment length in (D). The dotted box indicates the segment length of the posterior neighbouring segment during optogenetic stimulation. The arrow indicates that the neighbouring segment was returned to the equilibrium length just after the optogenetic stimulus was removed.

**Additional file 8: Fig. S8.** Frequency of crawling in a low friction condition. (A) Comparison of crawling frequency between larvae on agarose and floating. (B) Comparison of crawling frequency in simulation between in the optimized condition (Control) and the absence of friction (No friction).

## Data Availability

All data generated or analysed during this study are included in this article and its supplementary information files. The segment kinematics data generated in this study are available from the Figshare repository with the DOI of 10.6084/m9.figshare.19289594 [72].

## Abbreviations

CPGs: Central pattern generators
GFP: Green fluorescent protein
T: Thoracic
A: Abdominal
CNS: Central nervous system
SLS: Standard linear solid
CCD: Charge-coupled device
PDMS: Polydimethylsiloxane
ATR: All-trans-retinal
LED: Light-emitting diode
ChR2: Channelrhodopsin2
