# Supplementary figures and images for: A neuromechanical model for Drosophila larval crawling based on physical measurements
Source: BMC Biol. 2022 Jun 15;20:130. doi: 10.1186/s12915-022-01336-w (PMC9199175; doi:10.1186/s12915-022-01336-w)

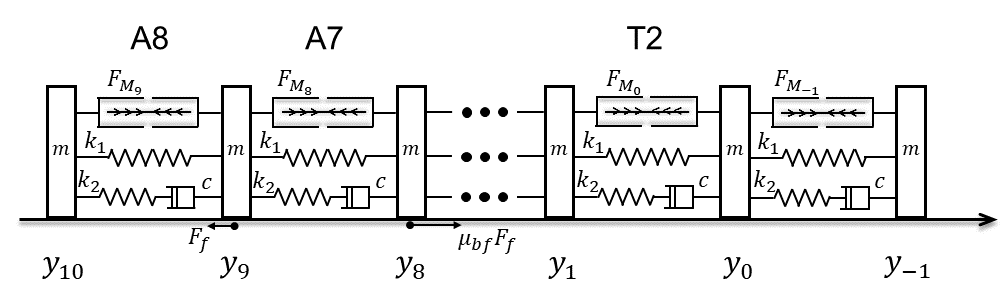

Supplement: Supplementary file 1 — Additional file 1: Fig. S1. A physical model with eleven segments. Based on the estimation of the number of segments, the fly larva was modelled by eleven segments. We assumed that y10 and y−1 were physically coupled. In the simulation results in this paper, segmental boundaries from y10 to y0 or segments from A8 to T2 were shown. [file 12915_2022_1336_MOESM1_ESM.png]

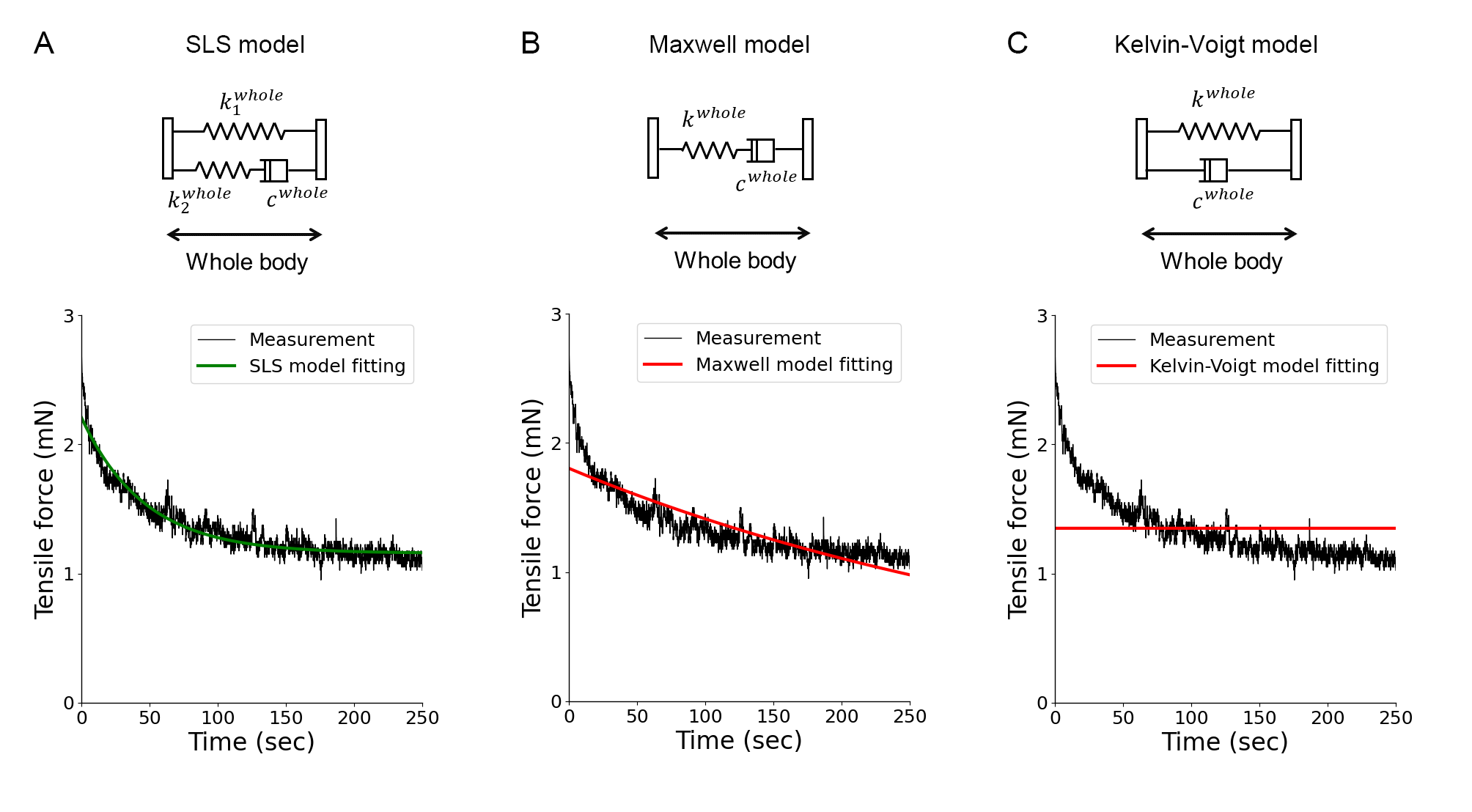

Supplement: Supplementary file 2 — Additional file 2: Fig. S2. Fitting stress-relaxation test data by three viscoelastic models. The same stress-relaxation test data was fitted with the SLS model (A), Maxwell model (B), and Kelvin-Voigt model (C). [file 12915_2022_1336_MOESM2_ESM.png]

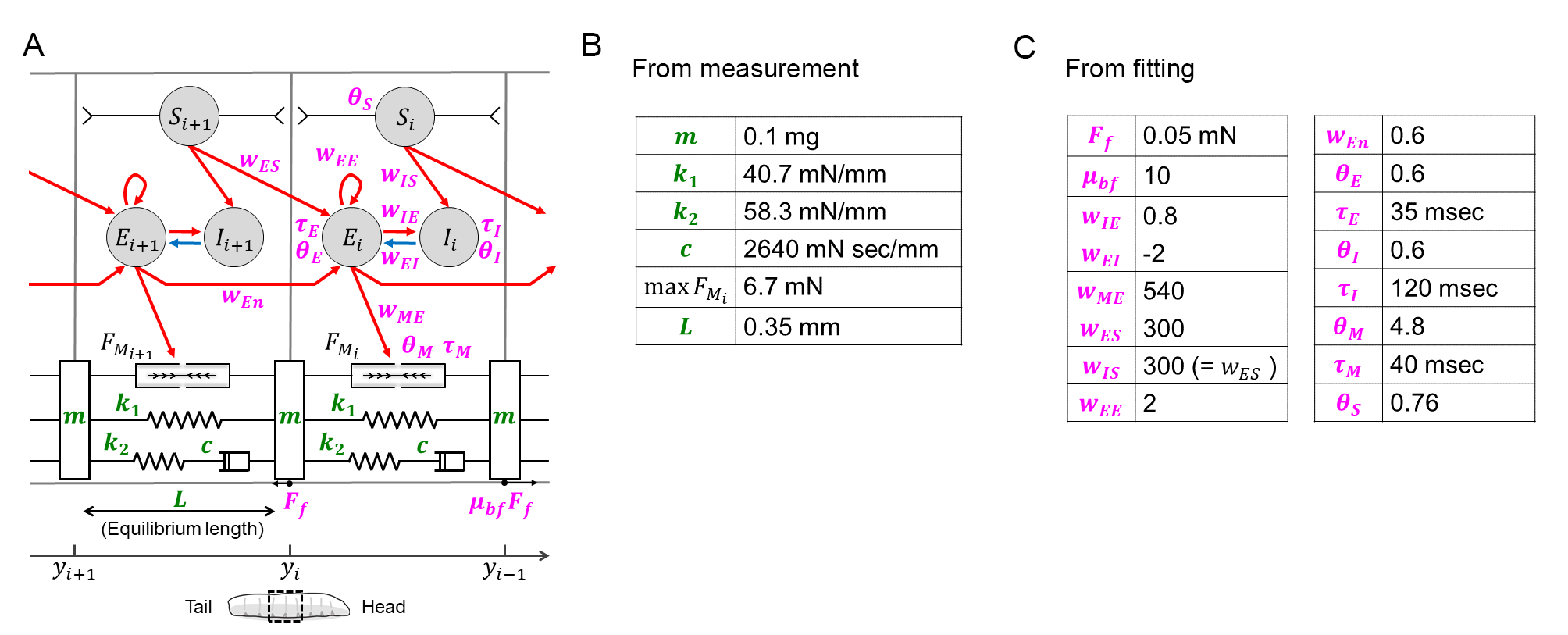

Supplement: Supplementary file 3 — Additional file 3: Fig. S3. Our physical model for larval crawling and its parameters. (A) Schematic of the physical model for larval crawling. (B) List of parameters whose values were obtained by measurement using fly larvae. (C) List of parameters whose values were obtained by fitting to reproduce larval crawling by the simulation. [file 12915_2022_1336_MOESM3_ESM.png]

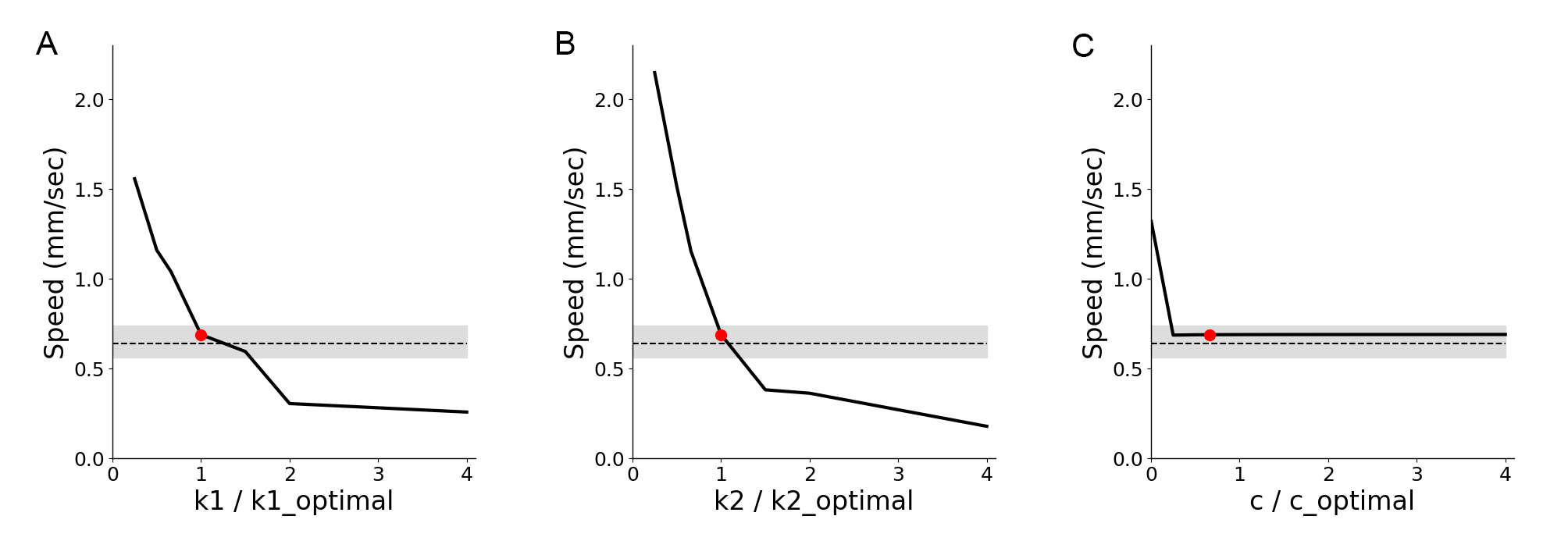

Supplement: Supplementary file 4 — Additional file 4: Fig. S4. Perturbation analysis on viscoelasticity. Plots of speed as viscoelasticity was perturbed. (A) Perturbation of the spring constant k1. (B) Perturbation of the spring constant k2. (C) Perturbation of the dumping coefficient c. The horizontal axes were normalized by the optimized values (k1 _ optimal, k2 _ optimal, and c _ optimal), respectively. Grey shaded regions show the range of speed observed in the experiment with third-instar larvae. Red dots indicate the optimized simulation condition. The minimum value in the horizontal axis in the plot of (C) equals 1/400, which corresponds to the value in Pehlevan et al. (2016) [10]. [file 12915_2022_1336_MOESM4_ESM.png]

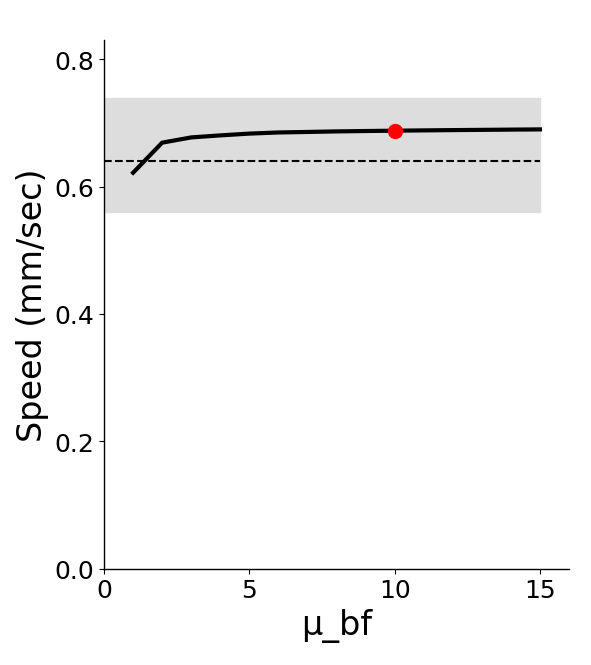

Supplement: Supplementary file 5 — Additional file 5: Fig. S5. Perturbation analysis on the asymmetricity in friction. Plots of speed as the friction asymmetricity was perturbed. The grey shaded region shows the range of speed observed in the experiment with third-instar larvae. The red dot indicates the optimized simulation condition. [file 12915_2022_1336_MOESM5_ESM.png]

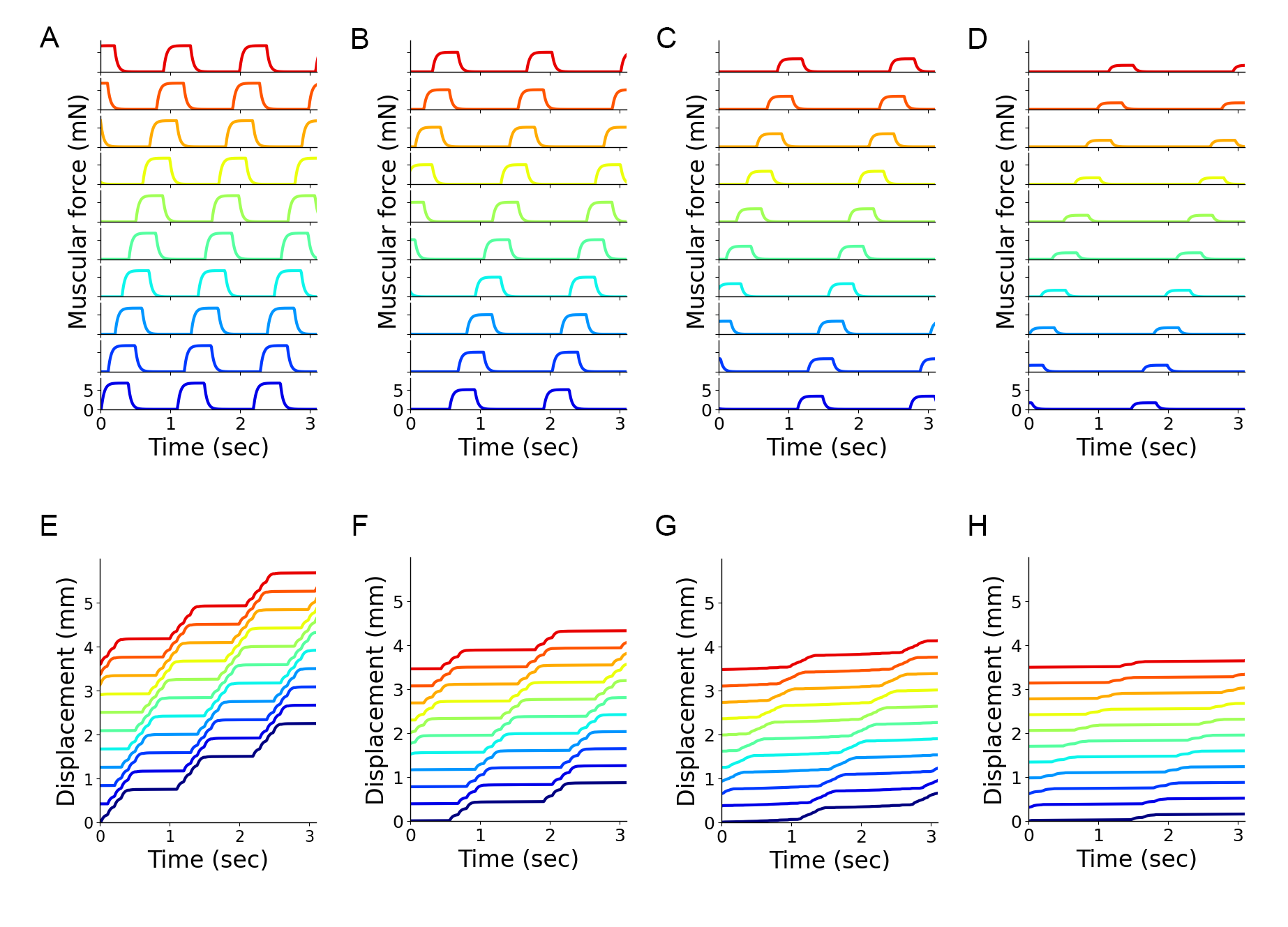

Supplement: Supplementary file 6 — Additional file 6: Fig. S6. Perturbation analysis on the maximum muscular force. Muscular force and segment displacement when the maximum muscular contraction force was perturbed. (A-D) Muscular forces in all the segments. Line colours correspond to those in Fig. 1B and C. (E-H) Displacement of the segmental boundaries. Line colours correspond to those in Fig. 1E and F. The ratio of the maximum contraction force to the optimized force: A and E, 100%; B and F, 75%; C and G, 50%; D and H, 25%. [file 12915_2022_1336_MOESM6_ESM.png]

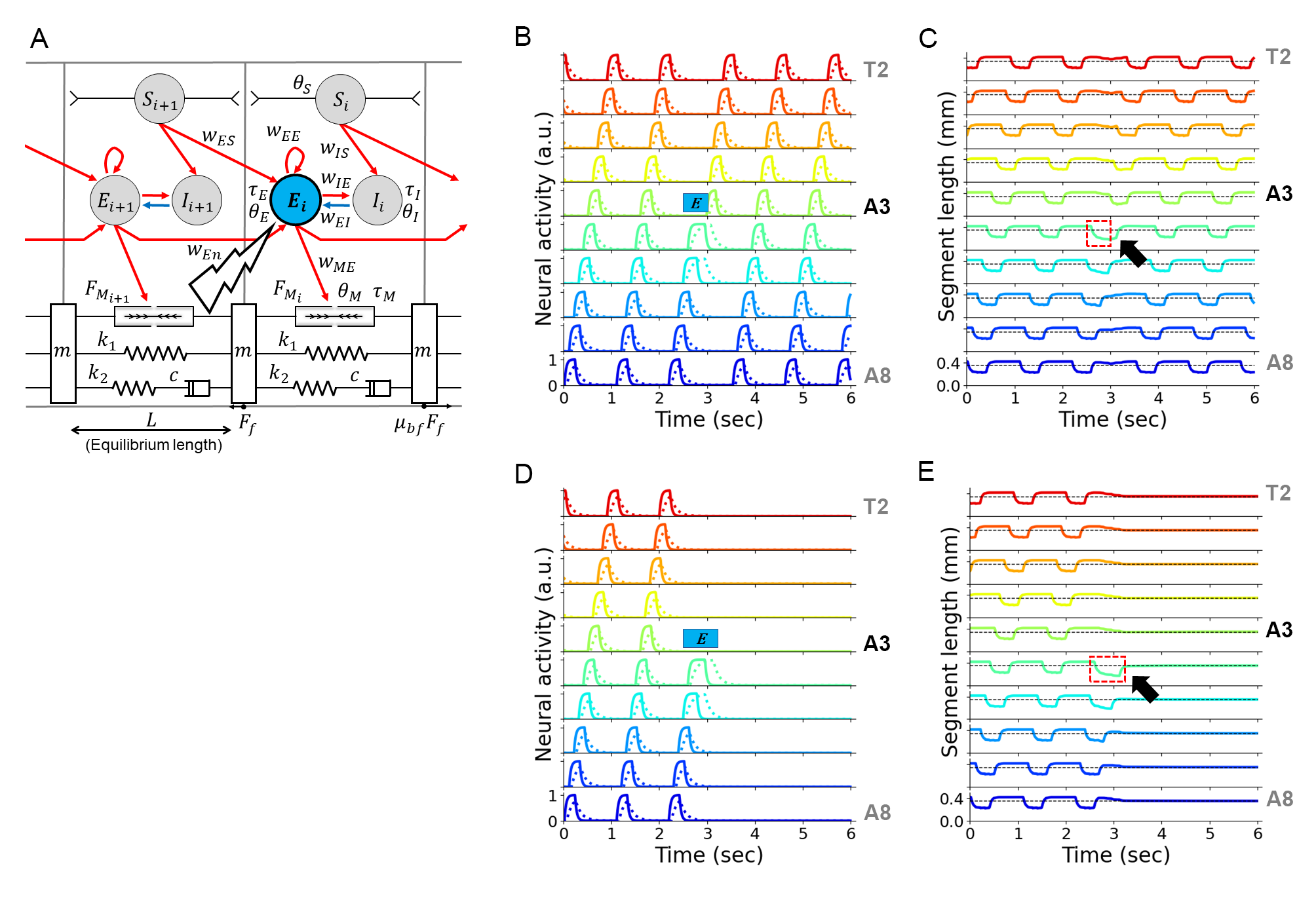

Supplement: Supplementary file 7 — Additional file 7: Fig. S7. Different durations of optogenetic stimulation in the simulation exhibit distinct phenotypes in the propagation of waves. (A) Schematic of optogenetic silencing of excitatory neurons in a single segment shown by a blue disk. (B) Neural activity with the optogenetic silencing of excitatory neurons. Excitatory neurons in the A3 segment were silenced for 0.5 seconds, marked by a blue bar. Waves were resumed just after the optogenetic silencing was removed. (C) Traces of segment length in (B). The dotted box shows the segment length of the posterior neighbouring segment during optogenetic stimulation. The arrow indicates that the neighbouring segment was contracted just after the optogenetic stimulus was removed. (D) Neural activity with the optogenetic silencing of excitatory neurons for 0.7 seconds. Excitatory neurons in the A3 segment were silenced as marked by a blue bar. Waves were arrested even after the optogenetic silencing was removed. (E) Traces of segment length in (D). The dotted box indicates the segment length of the posterior neighbouring segment during optogenetic stimulation. The arrow indicates that the neighbouring segment was returned to the equilibrium length just after the optogenetic stimulus was removed. [file 12915_2022_1336_MOESM7_ESM.png]

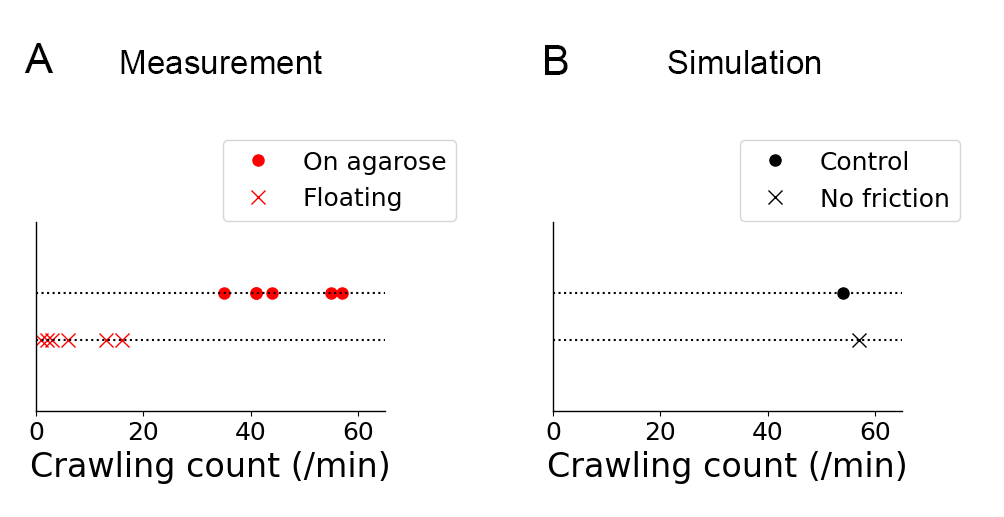

Supplement: Supplementary file 8 — Additional file 8: Fig. S8. Frequency of crawling in a low friction condition. (A) Comparison of crawling frequency between larvae on agarose and floating. (B) Comparison of crawling frequency in simulation between in the optimized condition (Control) and the absence of friction (No friction). [file 12915_2022_1336_MOESM8_ESM.png]
